# Effectiveness, acceptability, adherence, and safety of exergaming for depressive symptoms: a systematic review and meta-analysis

**DOI:** 10.1038/s41746-026-02479-8

**Published:** 2026-02-25

**Authors:** Di Tang, Chang Liu, Jinde Liu, Tong Liu, Ruisi Ma, Kim-wai Raymond Sum

**Affiliations:** 1 Department of Sports Science and Physical Education, Faculty of Education, The Chinese University of Hong Kong, Hong Kong, China; 2 The Nethersole School of Nursing, Faculty of Medicine, The Chinese University of Hong Kong, Hong Kong, China; 3 Vanke School of Public Health, Tsinghua University, Beijing, China; 4 Faculty of Physical Education, Fudan University, Shanghai, China; 5 School of Psychology and Mental Health, North China University of Science and Technology, Tangshan, China; 6 Faculty of Physical Education, Jinan University, Guangzhou, China

**Keywords:** Diseases, Health care, Medical research, Psychology, Psychology

## Abstract

Depression is a pervasive global disorder affecting 350 million people. Exergaming has emerged as a unique intervention for improving depressive symptoms by combining video games with physical exercise. However, existing evidence regarding its therapeutic effects remains inconsistent across studies. This systematic review and meta-analysis evaluated the effectiveness, safety, acceptability, adherence, and cost-effectiveness of exergaming interventions for depressive symptoms. We searched six major databases (PubMed, Cochrane Library, Scopus, PsycInfo, SPORTDiscus, and Web of Science) from inception to April 30, 2025, identifying 58 controlled trials involving 3614 participants. Using a multilevel random-effects model, we found that exergaming demonstrated a moderate, significant reduction in depression symptoms (*g* = −0.40, 95% CI: −0.56 to −0.25, *p* < 0.0001), with significant moderating effects observed for intervention frequency (showing larger effects for >3 times/week) and control group type (larger effects compared to no intervention/usual care). Additionally, a trend toward significance was found for age (*p* = 0.07), with larger effects observed in older adults (≥60 years). Furthermore, exergaming interventions showed high adherence rates, a good safety profile, and reasonable cost-effectiveness. Future larger-scale randomized controlled trials are needed to confirm these findings, alongside studies with extended follow-up periods to evaluate long-term sustainability.

## Introduction

Depression represents one of the most prevalent mental health disorders globally, exhibiting high prevalence rates across all age groups^1,2^. According to the World Health Organization, approximately 350 million people worldwide suffer from depressive disorders^3^. Depression not only significantly impairs patients’ quality of life but has also been demonstrated to exacerbate comorbid conditions, including cardiovascular diseases, anxiety disorders, and cancer^4–6^. The global burden of depression has further intensified during the COVID-19 pandemic, with a 27.6% increase in incidence^7^, emphasizing the critical need for accessible and effective interventions^8,9^.

Current primary treatment modalities for depression include psychotherapy and antidepressant medications^10,11^. Although research has substantiated the efficacy of both approaches in alleviating depressive symptoms^12,13^, both are subject to structural and clinical constraints. Psychotherapy typically entails high costs and is constrained by the limited availability of professional resources^14,15^. Meanwhile, pharmacological intervention, while remaining the principal treatment for moderate to severe depression^11,16^, requires strict medical supervision and may induce adverse effects, medication dependence, and withdrawal symptoms^17,18^. Furthermore, these traditional treatment modalities frequently encounter challenges regarding patient acceptance and long-term adherence^19,20^. From a global perspective, a significant treatment gap persists^21^, with research indicating that approximately two-thirds of adults with depression do not receive adequate care^22^. Consequently, the exploration of accessible, cost-effective, and patient-acceptable alternative therapeutic approaches has become increasingly imperative. For instance, emerging blended care models are attempting to overcome the limitations of traditional therapies^23,24^, while novel interventions—such as those leveraging physical activity and technology—are also being proposed and investigated^25^.

Within the current clinical landscape, physical exercise is increasingly recognized as an effective alternative intervention with minimal side effects, meeting the aforementioned criteria^26–29^. Clinical guidelines in the United Kingdom, the United States, and Australia have incorporated physical activity as a recommended adjunctive treatment for depression^10,30,31^. Extensive meta-analytic investigations have consistently demonstrated the significant therapeutic efficacy of conventional exercise for depressive disorders^26,27,29,32–34^. However, with the rapid advancement of digital technology, a novel form of exercise—exergaming—has become increasingly prevalent in daily activities and entertainment^35–38^. Exergames typically refer to interactive formats combining video games with physical activity, requiring participants to engage in bodily movement to participate in gameplay^39^. Compared to traditional exercise, exergames impose fewer spatial and environmental constraints while providing participants with real-time visual and auditory feedback through various technological means, significantly enhancing the interactivity and enjoyment of physical activity^36,40–42^.

Specifically, integrating game elements into physical exercise enhances enjoyment and entertainment value, particularly for children and adolescents who demonstrate a high preference for immersive experiences^36,43,44^. Furthermore, the incorporation of information technology provides real-time feedback and guidance through audiovisual-assisted instructional systems embedded within the games, while enabling precise personalization of difficulty levels and exercise intensity. This technological support effectively reduces barriers to participation and increases adaptability across diverse populations^45–48^. Such technology-enhanced design expands the potential user base, making exergames equally applicable to older adults and individuals with specialized rehabilitation needs, thereby offering accessible physical activity solutions for heterogeneous population groups^45,49,50^. Particularly during the COVID-19 pandemic, when social distancing measures restricted traditional exercise opportunities, the distinctive advantages of exergames became more pronounced, resulting in a substantial increase in their utilization^51,52^. Whether this novel form of exercise can achieve comparable therapeutic effects for depression as traditional exercise or demonstrate superior performance in terms of treatment efficacy, patient acceptance, and safety has garnered widespread attention among researchers.

Despite the increasing volume of research investigating the therapeutic efficacy of exergames for depression, these studies exhibit substantial heterogeneity in their findings^35^. Previously published systematic reviews and meta-analyses concerning exergames and depression also reflect certain limitations that warrant consideration. Many existing analyses have predominantly included studies published before 2019, potentially missing more recent developments in the field^35,53–57^, or have focused exclusively on specific populations, such as older adults or people with dementia^55,58,59^, or have conducted only systematic reviews and narrative syntheses without providing quantitative meta-analytic results^53,60^.

Notably, since 2019, particularly following the COVID-19 pandemic outbreak, exergames have undergone significant technological iterations and content expansion, with substantial advancements in prevalence, technological sophistication, and gameplay diversity, becoming a popular form of exercise and entertainment across age groups^61–63^. For instance, products such as Ring Fit Adventure for the Nintendo Switch, launched in 2019, have experienced surging sales^64,65^, while the introduction of immersive virtual reality (VR) technology has brought new developmental potential to exergames^66,67^. Concurrently, the post-pandemic popularization of exergames has generated considerable academic interest, with numerous relevant interventional studies published since 2019^68–78^, even exceeding the number of articles used for data synthesis in some previous meta-analyses. These recent studies potentially provide more robust and comprehensive evidence that may update conclusions drawn from previous meta-analyses.

Furthermore, the discrepancies across previous meta-analytic findings—ranging from significant and large effects^55^, to moderate^56^, minimal^79^, or even no effects^35^—likely originate from fundamental methodological differences and conceptual inconsistencies in study selection and analytical strategies. For instance, some meta-analyses have categorized virtual immersive experiences with minimal physical exertion^80^, non-gamified VR-based rehabilitation tools, virtual coaching programs^81^, or traditional non-electronic exercise games^82^ as exergames^48,55^, included studies where control groups also received traditional exercise interventions^55,56^, extensively incorporated single-arm studies without control groups into RCT research^35^, or failed to account for baseline differences between groups^79^. Additionally, when defining control groups, certain conditions labeled as “usual care” or “standardized care” may already include regular physical activity or exercise interventions, representing a fundamental difference from complete non-intervention groups or waitlist groups^72,83–85^, thereby increasing the complexity of result interpretation and potentially introducing bias in evaluating the effectiveness of the intervention^26,79,86^.

Given the substantial volume of newly published research, this systematic review and meta-analysis aims to update the assessment of exergames’ efficacy in reducing depression levels, systematically evaluate and analyze the heterogeneity in existing empirical research, and address the deficiencies of previous reviews. We conducted a stratified analysis based on the nature of control groups, assessing the differential effects of exergames compared to non-intervention groups, waitlist groups, non-exercise usual care control groups, and traditional exercise control groups, with the objective of providing more precise scientific evidence for clinical practice and future research.

Beyond intervention efficacy, we also systematically evaluated participant acceptability, adherence, and safety of exergaming, as well as whether cost-effectiveness analyses of exergaming could address the limitations of current traditional treatment approaches, potentially helping to resolve the problem of low treatment coverage rates for depression.

## Results

In total, 4369 articles were identified. After the screening process, 58 articles were included in the final analysis, with 50 articles retrieved from database searching and 8 additional articles identified through citation search. The complete selection process is illustrated in Supplementary Figure 1 (p. 23). The details of excluded studies at full-text screening are presented in the Supplementary Table 2 (pp. 7–8).

### Study characteristics

Among the 58 studies included in this analysis, sample sizes ranged from 10 to 769, with a total of 3614 participants. The mean age of participants ranged from 9.14 to 85.19 years. Twenty-three studies enrolled healthy participants^72,76,83,84,87–105^, 19 included patients with somatic disorders^68–71,75,77,78,106–117^, and 16 recruited patients with neurological or psychological disorders^73,74,118–131^. Geographically, 23 studies were conducted in Europe^69,73,74,76,77,83,87,90,92,95,108,109,114,116,117,120,121,127–130^, 21 in Asia^68,70–72,75,78,89,93,94,101,105,107,110–113,122–126,131^, 12 in the Americas^88,91,96–99,102,103,106,115,119^, and 2 in Oceania^100,104^. The study designs comprised 50 randomized controlled trials^68–75,77,78,87,90,91,93–98,100–109,111–131^, and 8 non-randomized controlled studies^76,83,84,88,89,92,99,110^. The publication years of included studies ranged from 2011 to 2025.

The exergaming interventions utilized four main categories of devices: Microsoft (e.g., X-box), Nintendo (e.g., Switch), professional rehabilitation devices, and other platforms (e.g., Sony PlayStation and custom-designed platforms). The intervention periods varied from 1 day to 1 year, with frequencies ranging from once to seven times per week, and individual session durations spanning 14.4 to 60 min. Depression was primarily assessed using validated instruments such as the Beck Depression Inventory (BDI), Hospital Anxiety and Depression Scale (HADS), and Geriatric Depression Scale (GDS). The control groups in the included studies comprised several categories. Usual care included various forms of standard treatments: conventional physical therapy, occupational therapy, physiotherapy, rehabilitation exercises, and nursing care. Notably, none of the control groups involved protocols explicitly designated as antidepressant medication or depression-specific psychotherapy as the comparator. Consequently, the provisional subgroup analysis for “traditional antidepressant treatment” was not conducted. Traditional physical exercise interventions consisted of balance training, resistance exercises, bicycle training, and yoga. No intervention groups either received no treatment or were assigned to a waiting list. Additional control conditions included educational sessions (health talks, memory workshops), recreational activities (board games, computer games, watching music videos). Detailed characteristics of the included studies are presented in Supplementary Table 4 (pp. 14–19). Risk-of-bias assessments and GRADE are presented in Supplementary Figures 2–5 (pp. 24–29) and Supplementary Table 6 (p. 22).

### Meta-analysis and moderator analyses

Our meta-analysis synthesizing evidence from 58 studies (*k* = 64) demonstrated that exergaming had a moderate effect on depression (*g* = −0.40, 95% CI, −0.56 to −0.25, *p* < .0001; Supplementary Fig. 6, p. 30). Total heterogeneity was observed (*I*^2^ = 77.42%), comprising both between-study (*I*^2^ = 64.42%) and within-study heterogeneity (*I*^2^ = 13%).

Categorical moderator analyses identified two significant moderators of exergaming effects on depression (Table 1). Specifically, exergaming with higher frequency (>3 times/week) showed larger effects (*g* = −0.69, 95% CI, −1.00 to −0.38) than those with lower frequency (≤3 times/week; *g* = −0.32, 95% CI, −0.50 to −0.14; *F*_1, 60_ = 4.16, *p* < .05). Additionally, compared to exercise control groups (*g* = −0.13; 95% CI, −0.33 to 0.08), exergaming showed significantly larger effects when compared to no intervention or usual care (*g* = −0.57, 95% CI, −0.74 to −0.40; *F*_1, 62_ = 13.60, *p* < 0.001). Notably, the effect of exergaming on reducing depressive symptoms was not significantly different from that of traditional physical exercise (*p* = 0.22). Furthermore, there was a marginally significant moderating effect of age (*F*_1, 62_ = 3.44, *p* = 0.07), with older adults (≥60 years) showing a tendency toward larger effects (*g* = −0.52, 95% CI, −0.72 to −0.32) compared to younger participants (<60 years; *g* = −0.23, 95% CI, −0.47 to 0.01). In the continuous moderator analyses, none of the examined variables significantly moderated the effects of exergaming on depression (Table 2).

**Table 1:** Categorical moderator analyses of exergaming effects on depression

Moderator	Categories	*k*	Hedges’ *g* (95% CI)	*F* statistic	*p*
Population	Healthy	25	−0.50 (−0.75 to −0.25)	*F*_2, 61_ = 0.56	0.58
	Neurological and psychological disorders	19	−0.39 (−0.70 to −0.08)		
	Somatic disorders	20	−0.30 (−0.58 to −0.02)		
Age, year	<60	26	−0.23 (−0.47 to 0.01)	*F*_1, 62_ = 3.44	0.07
	≥60	38	−0.52 (−0.72 to −0.32)		
Set	Clinical	39	−0.34 (−0.54 to −0.15)	*F*_1, 59_ = 0.12	0.73
	Field	22	−0.40 (−0.65 to −0.15)		
Frequency, times/week	≤3	45	−0.32 (−0.50 to −0.14)	*F*_1, 60_ = 4.16	<0.05
	>3	17	−0.69 (−1.00 to −0.38)		
Week	<8	30	−0.27 (−0.49 to −0.04)	*F*_1, 62_ = 2.81	0.10
	≥8	34	−0.53 (−0.74 to −0.31)		
Session length, min	<30	15	−0.28 (−0.57 to 0.01)	*F*_1, 59_ = 0.73	0.40
	≥30	46	−0.42 (−0.60 to −0.25)		
Device	Microsoft	27	−0.39 (−0.63 to −0.14)	*F*_3, 60_ = 0.72	0.54
	Nintendo	20	−0.41 (−0.70 to −0.12)		
	Medical/rehabilitation	9	−0.65 (−1.10 to −0.22)		
	Others	8	−0.22 (−0.63 to 0.19)		
Control	Exercise	25	−0.13 (−0.33 to 0.08)	*F*_1, 62_ = 13.60	<0.001
	No Intervention or usual care	39	−0.57 (−0.74 to −0.40)		
Country	Developed country	37	−0.35 (−0.56 to −0.14)	*F*_1, 62_ = 0.55	0.46
	Developing country	27	−0.47 (−0.71 to −0.23)		
Study design	NRCT	10	−0.38 (−0.78 to 0.02)	*F*_1, 62_ = 0.02	0.90
	RCT	54	−0.41 (−0.58 to −0.23)		
Scales	BDI	14	−0.56 (−0.91 to −0.20)	*F*_3, 60_ = 1.57	0.21
	GDS	16	−0.47 (−0.78 to −0.16)		
	HADS	16	−0.12 (−0.43 to −0.19)		
	Others	18	−0.49 (−0.77 to −0.21)		
Publication year	≤2019	22	−0.30 (−0.56 to −0.04)	*F*_1, 62_ = 1.03	0.31
	>2019	42	−0.46 (−0.66 to −0.27)		

*BDI* Beck Depression Inventory, *GDS* Geriatric Depression Scale, *HADS* Hospital Anxiety and Depression Scale, *RCT* Randomized controlled trial, *NRCT* Non-randomized controlled trial.

**Table 2:** Continuous moderator analyses of exergaming effects on depression

Moderator	Range	*k*	Intercept (95% CI) *	*β* (95% CI) ^#^	*F* statistic	*p*
Age, year	9.41–85.19	61	−0.09 (−0.56 to 0.39)	0.00 (−0.01 to 0.00)	*F*_1, 59_ = 1.45	0.23
Sex (male), %	0–91	60	−0.58 (−0.91 to −0.25)	0.39 (−0.33 to 1.12)	*F*_1, 58_ = 1.20	0.28
Week	0.86–52.14	63	−0.46 (−0.70 to −0.23)	0.01 (−0.01 to 0.03)	*F*_1, 61_ = 0.39	0.53
Frequency, times/week	1–7	62	−0.14 (−0.54 to 0.27)	−0.09 (−0.20 to 0.03)	*F*_1, 60_ = 2.32	0.13
Session length, min	14.4–60	61	−0.25 (−0.65 to 0.15)	0.00 (−0.01 to 0.01)	*F*_1, 59_ = 0.53	0.47
Total session, No.	37.5–3635	63	−0.33 (−0.63 to −0.03)	0.00 (−0.02 to 0.01)	*F*_1, 61_ = 0.43	0.51
Total length, min	1–64.3	62	−0.28 (−0.52 to −0.05)	0.00 (0.00 to 0.00)	*F*_1, 59_ = 1.08	0.30

*Intercept indicates the estimated effect size when the moderator was 0.

^#^*β* indicates the change in effect size associated with increasing the value of the continuous moderator by 1.

### Sensitivity analysis and publication bias

One outlier was detected for the overall effect on depression^71^. After removing the outlier, the sensitivity analysis confirmed the robustness of our findings, with exergaming maintaining a significant moderate effect on depressive symptoms (*g* = −0.37, 95% CI, −0.51 to −0.22, *p* < 0.0001).

**Fig. 1:**
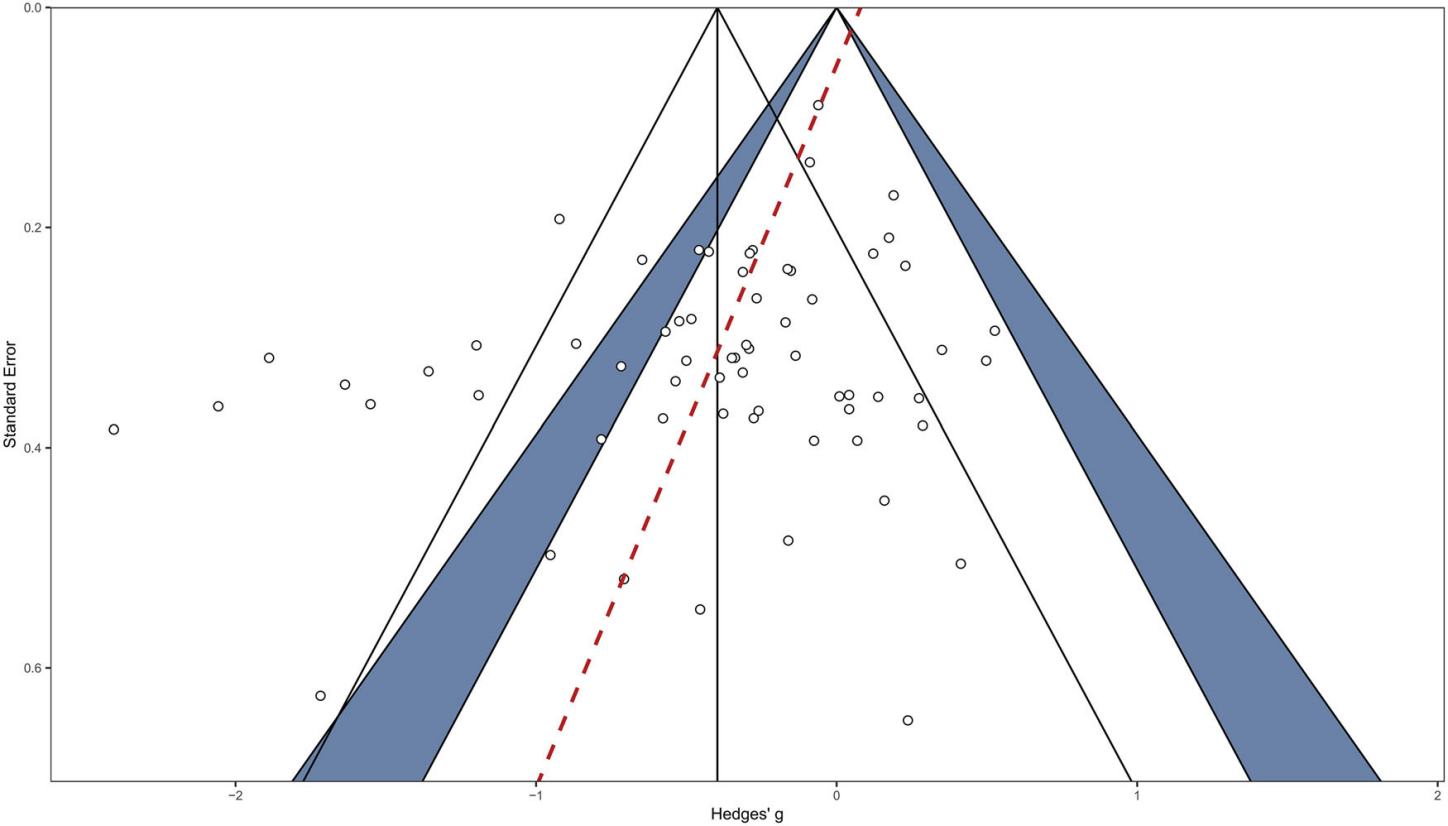
Significance and confidence contours–enhanced funnel plot. Significance contours at 0.05 and 0.01 levels are noted by the blue shaded area. This figure was generated using R version 4.4.2.

**Fig. 2:**
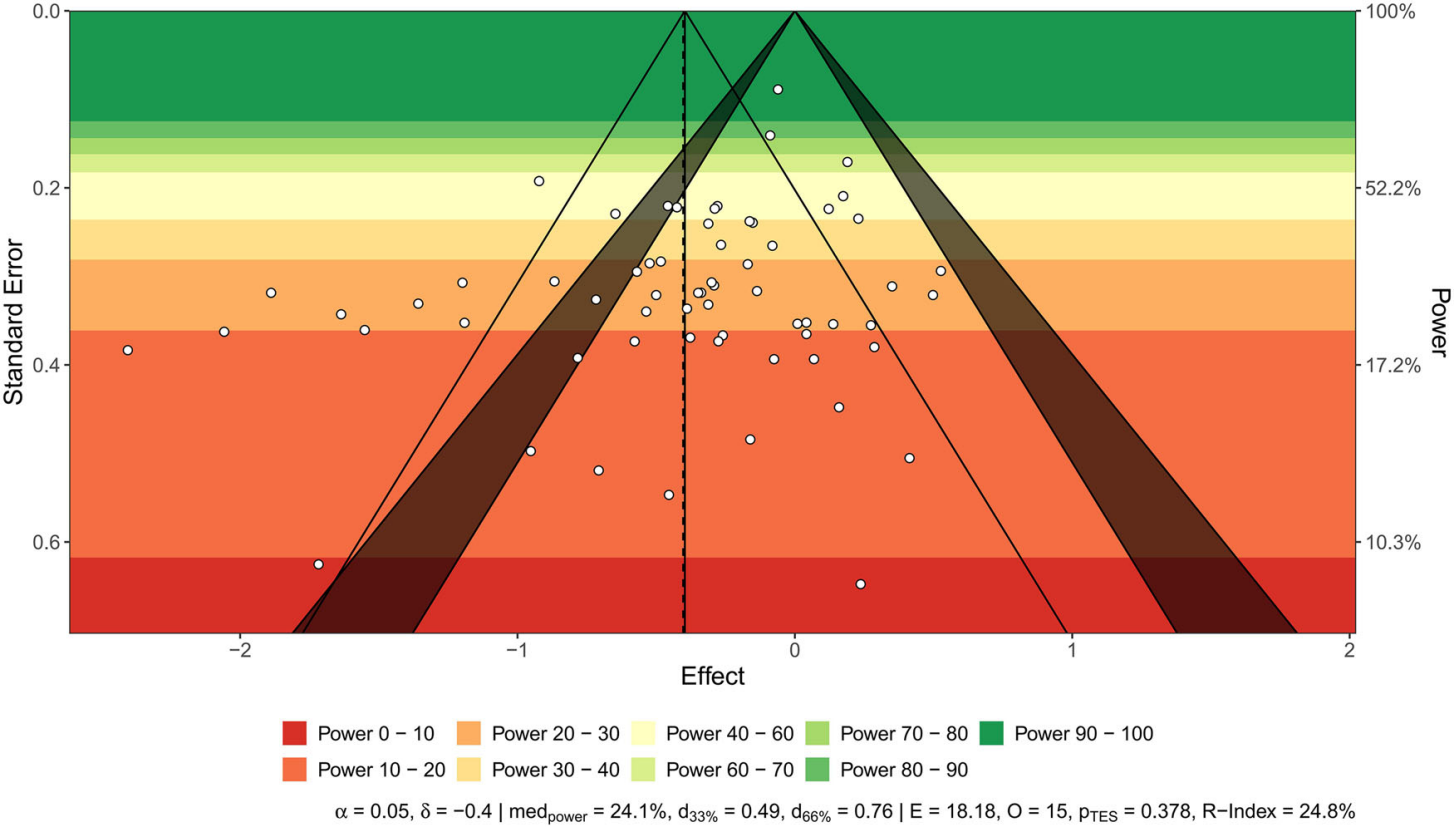
Sunset funnel plot. Significance contours at 0.05 and 0.01 levels are noted by the shaded area. d_33%_ and d_66%_ represent the true effect sizes required to obtain 33% and 66% median power levels. The indicators E, O, and PTES display the excess significance test outcomes. The R-index measures the predicted reproducibility of the study findings. This figure was generated using R version 4.4.2.

The funnel plot showed a relatively symmetric distribution of studies around the overall effect size, and Egger’s test confirmed no significant asymmetry (*F*_1, 62_ = 0.41, *p* = 0.53; Fig. 1). The sunset funnel plot indicated a median power of 24.1%. The probability of replicating these studies was 24.8% (Fig. 2).

### Adverse events, adherence, acceptance, and cost

Among the included studies, 27 studies monitored adverse events^68,70,73,74,88,91,99–101,104,107,109,111,112,114–116,118,120,121,123,125,127–131^, 10 studies measured participants’ acceptability and satisfaction^73,74,83,91,101,104,114,118,120,128^, and 3 studies documented intervention costs^83,108,128^. Of the 27 studies monitoring adverse events, 20 reported no adverse events^68,70,73,88,100,101,107,109,111,112,114,118,120,123,125,127,129–132^, and 3 reported no serious adverse events^99,104,128^. Two studies each reported one adverse event^116,121^, with one being unrelated to the intervention. One study documented two adverse events^74^, while another study only indicated a “low incidence” without specific details^91^. Among the documented adverse events in the exergaming intervention groups, four serious adverse events were reported: one case of low back pain recurrence, one case of wrist fracture, one hospitalization for antiparkinsonian medication adjustment, and one case of diagnosed and treated chronic osteoarthritic knee pain.

Across 15 studies reporting adherence data, the reported metrics varied. 12 studies reported program adherence rates ranging from 65.7% to 100%, with an average of 89.1% (SD = 12.3%)^70,73,74,77,83,99,109,111,116,120,127,130^. Additionally, one study reported 23.1% (9/39) of participants completed at least two-thirds of the sessions^100^. Regarding achievement of prescribed doses, one 12-month study reported 50.8% of participants reached the minimum required training dose (80 min per week)^104^. One additional study reported comparable adherence between exergaming and traditional physical exercise interventions, without providing specific data^119^.

Ten studies measured participants’ acceptance or satisfaction with exergaming interventions, with all studies indicating positive attitudes and high acceptance towards exergaming^73,74,83,91,101,104,114,118,120,128^. Three studies conducted statistical calculations on the costs or cost-effectiveness of exergaming interventions. One reported that the incremental cost-effectiveness ratio (ICER) for Microsoft Kinect-based exergames intervention was £15,209.80 per quality-adjusted life year (QALY), with a 61%–73% probability of being cost-effective within the National Institute for Health and Care Excellence (NICE) threshold range (£20,000 to £30,000 per QALY)^83^. In terms of specific implementation costs, different projects showed varying cost structures: The Microsoft Kinect-based VirtualEx-FM intervention cost approximately €10,000 for groups of 3 participants over 8 weeks, including fixed costs of €6000 (software development and hardware) and operating costs of €4000 (technical staff and venue rental)^108^. The Nintendo Wii-based Mii-vitaliSe intervention cost £684 per person, comprising £384 for physiotherapy time (averaging 12 h at £32 per hour) and £300 for equipment (Nintendo Wii console and accessories)^128^. These findings generally demonstrate reasonable cost-effectiveness and practical feasibility.

## Discussion

This is the largest meta-analysis, synthesizing evidence from 58 studies to investigate the effects of exergaming on depression. Our results demonstrated that exergaming significantly improved depressive symptoms, which is consistent with previous findings^35,55,56,79^. This study addressed several limitations of existing systematic reviews and meta-analyses. By incorporating a substantial number of studies published after 2019, we significantly expanded the sample size and provided up-to-date evidence in this field. Methodologically, we employed a more rigorous approach to effect size calculation by using change scores from baseline, which effectively controlled for baseline differences between intervention and control groups. Furthermore, we conducted detailed subgroup analyses based on control group types, allowing for more precise effect size estimation. Notably, this study provided the first systematic summary of adverse events, adherence, acceptance, and cost associated with exergaming-based physical exercise. These methodological improvements enabled us to more accurately evaluate the clinical effectiveness of exergaming for depression, providing more reliable evidence for clinical practice.

When evaluating intervention effectiveness, multiple key factors warrant careful consideration. Our moderation analyses identified two significant moderating variables: intervention frequency and control group type. Specifically, high-frequency interventions (>3 sessions/week) demonstrated superior antidepressant effects compared to low-frequency interventions (≤3 sessions/week). This frequency-dependent effect may operate through its influence on intervention fidelity and effectiveness^133,134^. The underlying mechanism could be attributed to sustained exercise stimulation and its cumulative effects on neurophysiological adaptation^135,136^. Additionally, we found that exergaming intervention with a session duration <30 min showed non-significant effects. Therefore, a recommended intervention protocol would be more than three sessions per week, with each session lasting ≥30 min. Moreover, exergaming showed significantly larger effects when compared to no intervention or treatment as usual. Notably, no significant differences were observed between exergaming and traditional exercise interventions, suggesting that exergaming could serve as a viable alternative to conventional exercise programs. Although exergaming did not demonstrate superior antidepressant effects compared to traditional exercise, implementation feasibility is another crucial aspect in evaluating interventions. Data from included studies indicated that exergaming showed good adherence, with an average completion rate of 89.1%. Notably, a one-year intervention study revealed that the exergaming group maintained 80 min of weekly training throughout the year^104^. This high level of adherence is significant, especially considering that attrition rates in traditional exercise programs for older adults can be as high as 50%^137^. Therefore, the gaming component may serve as an effective strategy to enhance exercise adherence. The superior adherence observed in exergaming can be attributed to the deep restructuring of exercise behavior patterns via gamification, which aligns with the principles of Persuasive System Design (PSD)^138^ and the Technology Acceptance Model (TAM)^139^. First, exergames designed with gamification strategies effectively mitigate the issue of delayed rewards inherent in traditional exercise by establishing real-time feedback loops^140^. Consistent with the Self-monitoring and Reward principles of PSD, by instantly translating physical movements into in-game visual effects and scores, this mechanism transforms vague long-term health benefits into instant gratification, effectively bridging the motivational gap during exertion. The integration of VR and game elements enhances engagement through immediate feedback and enjoyment, while the convenience of home-based implementation further promotes participation^140,141^. Second, exergames reshape participants’ affective attitude by enhancing the hedonic value and ease of use of the activity^142–144^. The fun and challenge introduced by gamification successfully transform exercise from a “health-driven task” into “pleasure-driven play”, thereby significantly reducing psychological boredom. Furthermore, adaptive game difficulty, the convenience of home-based implementation, and detailed built-in interactive instructional guidance effectively lower the operational and technical barriers for users^145,146^. From the perspective of TAM, this dual enhancement of perceived ease of use and enjoyment increases user acceptance, thereby significantly promoting engagement and adherence to exercise^147,148^. These features collectively improve psychological mood and motivation, leading to sustained adherence. Research indicates that when the same cycling activity is gamified, participants exhibit significantly higher levels of positive affective attitude and adherence^149^. In addition, regarding participant satisfaction summarized in this study, all studies indicated that participants showed high levels of acceptance and satisfaction with exergaming. Based on these findings, exergaming shows potential as a long-term and home-based adjunctive therapeutic intervention. Safety and favorable cost-effectiveness data further support the clinical application of exergaming. Most studies reported no adverse events or only minor ones, with very few serious adverse events that were not necessarily related to the intervention. These findings support exergaming as a safe and economically viable intervention option.

Age may play another crucial role in intervention effectiveness. We observed a marginally significant age moderation effect, with adults aged 60 and above appearing to derive greater benefits. On one hand, older adults experience a decline in both cognitive and physical functioning^150,151^, and exergames have been proven to significantly improve these functions in the elderly. This improvement in daily life limitations may have a more sensitive impact on older adults’ emotional and psychological well-being compared to younger populations. Another possible explanation is the different depression causes across the age groups^35^. While younger adults’ depression often originates from work stress and relationship problems^152^, older adults’ depression is primarily linked to physical health decline and social isolation^153^. As exergames can improve both physical function and social interaction, they may be particularly effective in addressing depression among older adults. Furthermore, other reviews have also found that exergames have significant effects on balance and fall prevention in older adults^154–156^, and the elderly generally show good acceptance and satisfaction with these games^157^. Therefore, although exergames were initially designed as an entertainment medium for young people, given their various physical and psychological benefits for older adults, future development should focus on creating exergames specifically tailored to older adults’ lifestyle characteristics and health needs to maximize the health benefits of exergaming for this population.

Although our study aimed to address various limitations identified in previous meta-analyses and provide the most comprehensive and up-to-date evidence synthesis available, there are several limitations in our study that need to be acknowledged due to the constraints of included studies and the current quality of evidence. First, most of the included studies had low statistical power due to small sample sizes, which may affect the reliability and robustness of our meta-analytic findings. Second, the lack of follow-up data in most included studies limited our ability to assess the long-term effects of exergaming interventions on depression. This absence of long-term data makes it difficult to determine the sustained benefits of the intervention. Third, due to the nature of exergaming interventions, it was not possible to establish true placebo control conditions, which limited our ability to control for placebo effects. Finally, only a small number of studies reported data on adverse events, cost-effectiveness, acceptability, and adherence, and these outcomes were inconsistent across studies and calculated using varying methodologies. This heterogeneity in reporting and measurement methods prevented us from conducting a systematic quantitative synthesis of these important secondary outcomes, allowing only for narrative summaries and basic data compilation.

In conclusion, this systematic review and meta-analysis demonstrate that exergaming is an effective intervention for improving depression, showing favorable safety characteristics and high user adherence and acceptance. Our analysis suggests that an optimal intervention protocol consisting of sessions lasting at least 30 min, performed more than three times per week, may yield the most beneficial outcomes. However, to strengthen the evidence base and better understand the full therapeutic potential of exergaming interventions, future research should prioritize larger-scale randomized controlled trials with extended follow-up periods to evaluate the sustained effects of these interventions. Furthermore, the development of tailored exergaming interventions that specifically address the unique health needs and lifestyle considerations of older adults shows promising potential for maximizing the therapeutic benefits of this intervention approach.

## Methods

### Search strategy and selection criteria

We conducted this systematic review and meta-analysis following PRISMA guidelines^158^. The methodological framework for this systematic review and meta-analysis was pre-registered in PROSPERO (the International Prospective Register of Systematic Reviews) with the identifier CRD420251087148. Database searches were performed in PubMed, Cochrane Library, Scopus, PsycInfo, SPORTDiscus, and Web of Science from inception to April 30, 2025. The complete search strategy for each database is available in the Supplementary Note (pp. 2–4). Reference lists of relevant systematic reviews and meta-analyses were manually screened for additional eligible studies (Supplementary Table 1 pp. 5–6).

TTwo independent reviewers (DT and JL) screened titles and abstracts of identified records. Studies deemed potentially eligible underwent full-text assessment by the same reviewers. When insufficient information was available to determine eligibility, we contacted the corresponding authors for clarification. Discrepancies were resolved through discussion or adjudication by a third reviewer (CL) when necessary. Covidence was used for screening and data extraction processes.

Eligibility criteria and search strategy were established using the PICOS framework^159^. We included studies of any population to enable subsequent subgroup analyses and meta-regression examining differential intervention effects across demographic groups. Interventions needed to involve exergaming or virtual exercise delivered through VR technology, motion-sensing platforms, or other interactive systems requiring physical movement. Only randomized controlled trials or non-randomized controlled intervention studies with comparator groups (no treatment, usual care, conventional exercise, or waitlist) were eligible. We defined usual care as the prevailing standard medical or rehabilitation practices provided within the study context^160^. Although specific practices may vary by region, they all excluded the specific experimental intervention of interest (exergaming). Furthermore, if the usual care in the control group involved explicit, regularly performed traditional physical exercises, we classified it as the “traditional physical exercise” subgroup rather than the “usual care” group. Given our focus on depressive symptoms, we anticipated that the study population might include patients with moderate-to-severe depression. The usual care for such patients often involves antidepressant medication, psychotherapy, or counseling, which may differ significantly from the general usual care provided to other clinical populations in terms of its impact on depressive symptoms. Therefore, in cases where a control group received specific antidepressant medication or depression-specific psychotherapy, we also designated a “traditional antidepressant treatment” subgroup to analyze these separately. This distinction aimed to isolate the comparison between exergaming and traditional antidepressant treatment, separating it from comparisons involving general usual care or no intervention. Studies were required to assess depression using validated instruments at baseline and post-intervention. No restrictions were applied regarding intervention settings.

We excluded studies with interventions where technology merely provided immersive experiences without physical exercise requirements or gaming elements, or where imaging technology only simulated virtual environments for traditional exercise forms. Additionally, multimodal interventions in which exergaming represented just one component of a comprehensive treatment program were also excluded.

### Data analysis

Data extraction was also conducted by two independent reviewers (DT and JL). We extracted data including the authors of the study, publication year, country, sample size, participant characteristics, intervention modality and devices (including intervention frequency, total duration, and length of individual sessions), control group setup, values and measurement instruments for depression outcome, as well as adverse events, adherence, acceptance, intervention costs or cost-effectiveness statistics (when available).

The meta-analysis was conducted using a multilevel random-effects model with restricted maximum likelihood estimation, implemented through R software (version 4.3.2) with the metafor package. Hedges’ *g* was used for data synthesis to correct for small sample bias. Considering that some studies showed significant differences in baseline measurements, we used change scores (post-test minus pre-test) to calculate effect sizes, thereby mitigating the influence of baseline differences on our analysis results. Heterogeneity was assessed using *I*^2^ statistics, with variance distribution examined across three levels (sampling variance, within-study variance, and between-study variance) through multilevel meta-analysis, while 95% prediction intervals were calculated for pooled effect sizes.

Potential categorical moderators examined encompass participant-related factors (population type, age groups), intervention-specific parameters (exercise sets, session frequency, program duration, session length), technical aspects (device types), methodological elements (control conditions, study design), contextual factors (country), assessment tools (depression scales), and temporal characteristics (publication year). Continuous moderator analyses were conducted on demographic variables (age, male percentage) and intervention-related parameters (program duration, weekly frequency, session duration, total session count, and cumulative intervention time). Cochran’s Q, Akaike information criterion, and Bayesian information criterion were employed to evaluate model fitness.

Outlier detection employed a ±3SD criterion for sensitivity testing^161,162^. Publication bias assessment incorporated both a multilevel Egger regression approach^163^ and contour-enhanced funnel plots^164^. If significant publication bias was detected, we employed the trim and fill method to impute missing effect sizes and calculate an adjusted overall effect estimate. The assessment of statistical power and study replicability was conducted using the sunset funnel plot methodology^165^.

Due to the inclusion of both randomized and non-randomized controlled trials in this review, we employed two different assessment tools. The Risk of Bias in Randomized Trials (RoB 2) tool was used to evaluate randomized controlled trials, while the Risk of Bias in Non-randomized Studies of Interventions (ROBINS-I) was applied to non-randomized studies. Two independent reviewers (DL and JL) conducted the assessments. If disagreements occurred, a third reviewer (CL) was consulted to make the final decision.

The Grading of Recommendations, Assessment, Development and Evaluation (GRADE) approach was applied to assess the certainty of evidence, evaluating the overall confidence in our evidence synthesis based on risk of bias, inconsistency, indirectness, imprecision, and publication bias.

## Supplementary information

Supplementary information

## Data Availability

The datasets generated and/or analyzed during the current study, including the extracted study characteristics and outcome data used for meta-analyses, are available from the corresponding author upon reasonable request.
