# Supplementary material for: Effectiveness, acceptability, adherence, and safety of exergaming for depressive symptoms: a systematic review and meta-analysis
Source: NPJ Digit Med. 2026 Feb 25;9:279. doi: 10.1038/s41746-026-02479-8 (PMC13046954; doi:10.1038/s41746-026-02479-8)
Supplement: Supplementary file 1 — Supplementary information [file 41746_2026_2479_MOESM1_ESM.pdf]

Supplementary Information

Table of Content

Tables

Supplementary Note	Search strategy	pp. 2-4
Supplementary Table 1	List of studies for citation search	pp. 5-6
Supplementary Table 2	List of studies excluded at full-text screening stage	pp. 7-8
Supplementary Table 3	PRISMA checklist	pp. 9-13
Supplementary Table 4	Descriptive characteristics of included studies	pp. 14-19
Supplementary Table 5	Abbreviation list	pp. 20-21
Supplementary Table 6	Grading of Recommendations Assessment, Development and Evaluation	p. 22

Figures

Supplementary Figure 1	PRISMA selection flow diagram	p. 23
Supplementary Figure 2	Risk of bias assessment for each included randomized study	p. 24-26
Supplementary Figure 3	Summary of risk of bias assessment for all included randomized studies	p. 27
Supplementary Figure 4	Risk of bias assessment for each included non-randomized study	p. 28
Supplementary Figure 5	Summary of risk of bias assessment for all included non-randomized studies	p. 29
Supplementary Figure 6	Forest plot for the effect of exergaming for depression	p. 30

Supplementary Note Search strategy

Web of Science (648)

((TS=(exergam* OR “exercise gam*” OR “active video gam*” OR “motion sensing game” OR “serious gam*” OR “motion gam*” OR “fitness gam*” OR “movement-based gam*” OR “active gam*” OR “motion-based gam*” OR “movement-based gam*” OR “gamified exercise” OR “virtual sport*” OR “digital sport*” OR “cyber sport*” OR “electronic sport*” OR esports OR e-sports OR “virtual realit*” OR “virtual-realit*” OR “VR-based intervention” OR “extended reality” OR Wii OR “X-box” OR XBox OR Kinect OR Nintendo OR Switch OR Playstation OR “Ring Fit Adventure” OR “video gam*” OR Zwift OR myWhoosh OR Rouvy OR “virtual running” OR “virtual cycling” OR TrainerRoad)) AND TS=(depress* OR dysthymia)) AND TS=(“randomized controlled trial*” OR “clinical trial*”)

Scopus (1226)

(TITLE-ABS-KEY (exergam* OR "exercise gam*" OR "active video gam*" OR "motion sensing game" OR "serious gam*" OR "motion gam*" OR "fitness gam*" OR "movement-based gam*" OR "active gam*" OR "motion-based gam*" OR "movement-based gam*" OR "gamified exercise" OR "virtual sport*" OR "digital sport*" OR "cyber sport*" OR “electronic sport*” OR esports OR e-sports OR "virtual realit*" OR "virtual-realit*" OR "VR-based intervention" OR "extended reality" OR Wii OR "X-box" OR xbox OR kinect OR nintendo OR switch OR playstation OR "Ring Fit Adventure" OR "video gam*" OR Zwift OR myWhoosh OR Rouvy OR "virtual running" OR "virtual cycling" OR TrainerRoad) AND TITLE-ABS-KEY (depress* OR dysthymia) AND TITLE-ABS-KEY ("randomized controlled trial*" OR "clinical trial*")) AND (EXCLUDE (DOCTYPE , "re") OR EXCLUDE (DOCTYPE , "le") OR EXCLUDE (DOCTYPE , "ed") OR EXCLUDE (DOCTYPE , "sh") OR EXCLUDE (DOCTYPE , "no") OR EXCLUDE (DOCTYPE , "er") OR EXCLUDE (DOCTYPE , "tb")) AND (LIMIT-TO (LANGUAGE , "English") OR LIMIT-TO (LANGUAGE , "Chinese"))

SportDiscus (15)

(exergam* OR "exercise gam*" OR "active video gam*" OR "motion sensing game" OR "serious gam*" OR "motion gam*" OR "fitness gam*" OR "movement-based gam*" OR "active gam*" OR "motion-based gam*" OR "movement-based gam*" OR "gamified exercise" OR "virtual sport*" OR "digital sport*" OR "cyber sport*" OR “electronic sport*” OR esports OR e-sports OR"virtual realit*" OR "virtual-realit*" OR "VR-based intervention" OR "extended reality" OR Wii OR X-box OR XBox OR Kinect OR Nintendo OR Switch OR Playstation OR "Ring Fit Adventure" OR "video gam*" OR Zwift OR myWhoosh OR Rouvy OR "virtual running" OR "virtual cycling" OR TrainerRoad) AND (depress* OR dysthymia) AND ("randomized controlled trial*" OR "clinical trial*")

Psycinfo (724)

#1 ((exergam* or "exercise gam*" or "active video gam*" or "motion sensing game" or "serious

gam*" or "motion gam*" or "fitness gam*" or "movement-based gam*" or "active gam*" or "motion-based gam*" or "movement-based gam*" or "gamified exercise" or "virtual sport*" or "digital sport*" or "cyber sport*" or “electronic sport*” or esports or e-sports or "virtual realit*" or "virtual-realit*" or "VR-based intervention" or "extended reality" or Wii or "X-box" or XBox or Kinect or Nintendo or Switch or Playstation or "Ring Fit Adventure" or "video gam*" or Zwift or myWhoosh or Rouvy or "virtual running" or "virtual cycling" OR TrainerRoad) and (depress* or dysthymia)).ti. and ("randomized controlled trial*" or "clinical trial*").af.

#2 ((exergam* or "exercise gam*" or "active video gam*" or "motion sensing game" or "serious gam*" or "motion gam*" or "fitness gam*" or "movement-based gam*" or "active gam*" or "motion-based gam*" or "movement-based gam*" or "gamified exercise" or "virtual sport*" or "digital sport*" or "cyber sport*" or “electronic sport*” or esports or e-sports or "virtual realit*" or "virtual-realit*" or "VR-based intervention" or "extended reality" or "Wii" or "X-box" or XBox or Kinect or Nintendo or Switch or Playstation or "Ring Fit Adventure" or "video gam*" or Zwift or myWhoosh or Rouvy or "virtual running" or "virtual cycling" OR TrainerRoad) and (depress* or dysthymia)).ab. and ("randomized controlled trial*" or "clinical trial*").af.

#1 OR #2

PubMed (457)

(("exergam*"[Title/Abstract] OR "exercise gam*"[Title/Abstract] OR "active video gam*"[Title/Abstract] OR "motion sensing game"[Title/Abstract] OR "serious gam*"[Title/Abstract] OR "motion gam*"[Title/Abstract] OR "fitness gam*"[Title/Abstract] OR "movement based gam*"[Title/Abstract] OR "active gam*"[Title/Abstract] OR "motion based gam*"[Title/Abstract] OR "movement based gam*"[Title/Abstract] OR "gamified exercise"[Title/Abstract] OR "virtual sport*"[Title/Abstract] OR "digital sport*"[Title/Abstract] OR "cyber sport*"[Title/Abstract] OR "electronic sport*"[Title/Abstract] OR "esports"[Title/Abstract] OR "e-sports"[Title/Abstract] OR "virtual realit*"[Title/Abstract] OR "virtual realit*"[Title/Abstract] OR "VR-based intervention"[Title/Abstract] OR "extended reality"[Title/Abstract] OR "Wii"[Title/Abstract] OR "X-box"[Title/Abstract] OR "XBox"[Title/Abstract] OR "Kinect"[Title/Abstract] OR "Nintendo"[Title/Abstract] OR "Switch"[Title/Abstract] OR "Playstation"[Title/Abstract] OR "Ring Fit Adventure"[Title/Abstract] OR "video gam*"[Title/Abstract] OR "Zwift"[Title/Abstract] OR "myWhoosh"[Title/Abstract] OR "Rouvy"[Title/Abstract] OR "virtual running"[Title/Abstract] OR "virtual cycling"[Title/Abstract] OR "TrainerRoad"[Title/Abstract]) AND ("depress*"[Title/Abstract] OR "dysthymia"[Title/Abstract]) AND ("randomized controlled trial*"[Title/Abstract] OR "clinical trial*"[Title/Abstract])) AND (chinese[Filter] OR english[Filter])

Cochrane Library (1299)

(exergam* OR (exercise NEXT gam*) OR (active NEXT video NEXT gam*) OR (motion NEXT sensing NEXT game) OR (serious NEXT gam*) OR (motion NEXT gam*) OR (fitness NEXT gam*) OR (movement-based NEXT gam*) OR (active NEXT gam*) OR (motion-based NEXT

gam*) OR (movement-based NEXT gam*) OR (gamified NEXT exercise) OR (virtual NEXT sport*) OR (digital NEXT sport*) OR (cyber NEXT sport*) OR (electronic NEXT sport*) OR esports OR e-sports OR (virtual NEXT realit*) OR (virtual-realit*) OR (VR-based NEXT intervention) OR (extended NEXT reality) OR Wii OR X-box OR XBox OR Kinect OR Nintendo OR Switch OR Playstation OR (Ring NEXT Fit NEXT Adventure) OR (video NEXT gam*) OR Zwift OR myWhoosh OR Rouvy OR (virtual NEXT running) OR (virtual NEXT cycling) OR TrainerRoad):ti,ab,kw AND (depress* OR dysthymia):ti,ab,kw AND ((randomized NEXT controlled NEXT trial*) OR (clinical NEXT trial*)):ti,ab,kw (Word variations have been searched)

Supplementary Table 1 List of studies for citation search

**1**	Li, J., Theng, Y. L., & Foo, S. (2016). Effect of exergames on depression: a systematic review and meta-analysis. Cyberpsychology, Behavior, and Social Networking, 19(1), 34-42.
**2**	Lee, S., Kim, W., Park, T., & Peng, W. (2017). The psychological effects of playing exergames: A systematic review. Cyberpsychology, Behavior, and Social Networking, 20(9), 513-532.
**3**	Andrade, A., Correia, C. K., & Coimbra, D. R. (2019). The psychological effects of exergames for children and adolescents with obesity: a systematic review and meta-analysis. Cyberpsychology, Behavior, and Social Networking, 22(11), 724-735.
**4**	Huang, K., Zhao, Y., He, R., Zhong, T., Yang, H., Chen, Y., ... & Chen, L. (2022). Exergame-based exercise training for depressive symptoms in adults: A systematic review and meta-analysis. Psychology of Sport and Exercise, 63, 102266.
**5**	Yen, H. Y., & Chiu, H. L. (2021). Virtual reality exergames for improving older adults' cognition and depression: a systematic review and meta-analysis of randomized control trials. Journal of the American Medical Directors Association, 22(5), 995-1002.
**6**	Cheung, D. S. K., Tse, H. Y. J., Wong, D. W. C., Chan, C. Y., Wan, W. L., Chu, K. K., ... & Ho, K. H. M. (2025). The Effects of Exergaming on the Depressive Symptoms of People With Dementia: A Systematic Review and Meta-Analysis. Journal of clinical nursing, 34(5), 1648-1664.
**7**	Abd-Alrazaq, A., Al-Jafar, E., Alajlani, M., Toro, C., Alhuwail, D., Ahmed, A., ... & Househ, M. (2022). The effectiveness of serious games for alleviating depression: systematic review and meta-analysis. JMIR Serious Games, 10(1), e32331.
**8**	Peng, Y., Wang, Y., Zhang, L., Zhang, Y., Sha, L., Dong, J., & He, Y. (2024). Virtual reality exergames for improving physical function, cognition and depression among older nursing home residents: A systematic review and meta-analysis. Geriatric nursing, 57, 31-44.
**9**	Chen, X., Wu, L., Feng, H., Ning, H., Wu, S., Hu, M., ... & Liu, X. (2023). Comparison of exergames versus conventional exercises on the health benefits of older adults: Systematic review with meta-analysis of randomized controlled trials. JMIR Serious Games, 11, e42374.
**10**	Kim, Y., Hong, S., & Choi, M. (2022). Effects of serious games on depression in older adults: systematic review and meta-analysis of randomized controlled trials. Journal of medical Internet research, 24(9), e37753.
**11**	Kashi, S. K., & Saatchian, V. (2025). Effectiveness of Exergame Intervention on Depressive Symptoms, Daily Living Activities, and Fear of Falling in Older Adults: A Systematic Review and Meta-Analysis of Randomized Controlled Trials. Clinical Gerontologist, 1-15.
**12**	Shi, X., Zhang, J., Wang, H., & Luximon, Y. (2024). The effectiveness of digital interactive intervention on reducing older adults' depressive and anxiety symptoms: a systematic review and meta-analysis. Gerontology, 70(9), 991-1011.
**13**	Townsend, C., Humpston, C., Rogers, J., Goodyear, V., Lavis, A., & Michail, M. (2022). The effectiveness of gaming interventions for depression and anxiety in young people: systematic review and meta-analysis. BJPsych Open, 8(1), e25.

14 Chen, P. J., Hsu, H. F., Chen, K. M., & Belcastro, F. (2023). VR exergame interventions among older adults living in long-term care facilities: A systematic review with Meta-analysis. Annals of physical and rehabilitation medicine, 66(3), 101702.

15 Chan, J. Y., Liu, J., Chan, A. T., & Tsoi, K. K. (2024). Exergaming and cognitive functions in people with mild cognitive impairment and dementia: a meta-analysis. npj Digital Medicine, 7(1), 154.

16 Wu, J., Xu, Z., Liu, H., Chen, X., Huang, L., Shi, Q., ... & Peng, L. (2023). Effects of commercial exergames and conventional exercises on improving executive functions in children and adolescents: Meta-Analysis of randomized controlled trials. JMIR Serious Games, 11, e42697.

Supplementary Table 2 List of studies excluded at full-text screening stage

Study	Title	Reason for exclusion
Ambron 2021	Virtual reality treatment displaying the missing leg improves phantom limb pain: a small clinical trial	Inappropriate design
Appel 2021	Vrct: randomized controlled trial evaluating the impact of virtual reality-therapy on BPSD and QOL of acute care in-patients with dementia	Incomplete data reports
Barsasella 2021	Effects of virtual reality sessions on the quality of life, happiness, and functional fitness among the older people: a randomized controlled trial from taiwan	Incomplete data reports
Bove 2019	A video game-based digital therapeutic to improve processing speed in people with multiple sclerosis: a feasibility study	Inappropriate design
Cano 2024	A multimodal group-based immersive virtual reality intervention for improving cognition and mental health in patients with post-covid-19 condition. A quasi-experimental design study	Non-target intervention
Chao 2024	The efficacy of early rehabilitation combined with virtual reality training in patients with first-time acute stroke: a randomized controlled trial	Non-target intervention
Choi 2016	Mobile game-based virtual reality rehabilitation program for upper limb dysfunction after ischemic stroke	Incomplete data reports
Cutter 2014	A pilot trial of a video game-based exercise program for methadone maintained patients	Missing outcome
Drazich 2023	Motivating older adults through immersive virtual exercise (MOTIVE): A randomized pilot study	Non-target intervention
Faruki 2022	Virtual reality immersion compared to monitored anesthesia care for hand surgery: A randomized controlled trial	Non-target intervention
Galperin 2023	Treadmill training with virtual reality to enhance gait and cognitive function among people with multiple sclerosis: A randomized controlled trial	Non-target intervention
Hortobágyi 2022	Comparative effectiveness of 4 exercise interventions followed by 2 years of exercise maintenance in multiple sclerosis: a randomized controlled trial	Duplicate reports
Jóźwik 2021	Evaluation of the impact of virtual reality-enhanced cardiac rehabilitation on depressive and anxiety symptoms in patients with coronary artery disease: A randomised controlled trial	Non-target intervention
Jóźwik 2021	The use of virtual therapy in cardiac rehabilitation of female patients with heart disease	Non-target intervention
Kempf 2013	Autonomous exercise game use improves metabolic control and quality of life in type 2 diabetes patients - a randomized controlled trial	Incomplete data reports
Lewandowski 2021	The use of virtual reality to reduce stress among inflammatory bowel disease patients treated with Vedolizumab	Non-target intervention
Li 2016	Exergames for older adults with subthreshold depression: Does higher playfulness lead to better improvement in depression?	Inappropriate design
Li 2016	Exergames vs. traditional exercise: Investigating the influencing mechanism of platform effect on subthreshold depression among older adults	Inappropriate design
Li 2024	Effects of virtual reality therapy for patients with breast cancer during chemotherapy: randomized controlled trial	Non-target intervention
Lier 2024	Virtual reality for postsurgical pain management: An explorative randomized controlled study	Non-target intervention
Liu 2020	Integrating virtual reality as distraction analgesia for office-based laryngology procedures	Missing outcome

Study	Title	**Reason for exclusion**
Ochi 2024	Exercising with virtual reality is potentially better for the working memory and positive mood than cycling alone	Non-target intervention
Pavlou 2012	The effect of virtual reality on visual vertigo symptoms in patients with peripheral vestibular dysfunction: A pilot study	Non-target intervention
Qi 2021	Acute VR competitive cycling exercise enhanced cortical activations and brain functional network efficiency in MA-dependent individuals	Non-target intervention
Qiu 2024	Application of virtual reality to enhance therapeutic Tai Chi for depression in elderly people	Non-target intervention
Rutkowski 2020	Effectiveness of an inpatient virtual reality-based pulmonary rehabilitation program among covid-19 patients on symptoms of anxiety, depression and quality of life: preliminary results from a randomized controlled trial	Non-target intervention
Rutkowski 2021	Evaluation of the efficacy of immersive virtual reality therapy as a method supporting pulmonary rehabilitation: a randomized controlled trial	Non-target intervention
Seo 2023	Virtual reality exercise program effects on body mass index, depression, exercise fun and exercise immersion in overweight middle-aged women: a randomized controlled trial	Non-target intervention
Syed 2021	Video game-based and conventional therapies in patients of neurological deficits: An experimental study	Incomplete data reports
Trost 2022	Immersive interactive virtual walking reduces neuropathic pain in spinal cord injury: Findings from a preliminary investigation of feasibility and clinical efficacy	Non-target intervention
Yu 2015	Effects of a physical activity program using exergame with elderly women	Ineligible language
Yuen 2011	Using Wii Fit to reduce fatigue among African American women with systemic lupus erythematosus: A pilot study	Inappropriate design
Zaitsu 2015	Association between extraversion and exercise performance among elderly persons receiving a video game intervention	Inappropriate design

Supplementary Table 3 PRISMA checklist

Section and Topic	Item #	Checklist item	Location where item is reported
**TITLE**			
Title	1	Identify the report as a systematic review.	1
**ABSTRACT**			
Abstract	2	See the PRISMA 2020 for Abstracts checklist.	2
**INTRODUCTION**			
Rationale	3	Describe the rationale for the review in the context of existing knowledge.	3-5
Objectives	4	Provide an explicit statement of the objective(s) or question(s) the review addresses.	3-5
**METHODS**			
Eligibility criteria	5	Specify the inclusion and exclusion criteria for the review and how studies were grouped for the syntheses.	9-10
Information sources	6	Specify all databases, registers, websites, organisations, reference lists and other sources searched or consulted to identify studies. Specify the date when each source was last searched or consulted.	9-10
Search strategy	7	Present the full search strategies for all databases, registers and websites, including any filters and limits used.	9 and supplementary note
Selection process	8	Specify the methods used to decide whether a study met the inclusion criteria of the review, including how many reviewers screened each record and each report retrieved, whether they worked independently, and if applicable, details of automation tools used in the process.	9-10

Section and Topic	Item #	Checklist item	Location where item is reported
Data collection process	9	Specify the methods used to collect data from reports, including how many reviewers collected data from each report, whether they worked independently, any processes for obtaining or confirming data from study investigators, and if applicable, details of automation tools used in the process.	9-10
Data items	10a	List and define all outcomes for which data were sought. Specify whether all results that were compatible with each outcome domain in each study were sought (e.g. for all measures, time points, analyses), and if not, the methods used to decide which results to collect.	9-10
	10b	List and define all other variables for which data were sought (e.g. participant and intervention characteristics, funding sources). Describe any assumptions made about any missing or unclear information.	9-10
Study risk of bias assessment	11	Specify the methods used to assess risk of bias in the included studies, including details of the tool(s) used, how many reviewers assessed each study and whether they worked independently, and if applicable, details of automation tools used in the process.	10-11
Effect measures	12	Specify for each outcome the effect measure(s) (e.g. risk ratio, mean difference) used in the synthesis or presentation of results.	10-11
Synthesis methods	13a	Describe the processes used to decide which studies were eligible for each synthesis (e.g. tabulating the study intervention characteristics and comparing against the planned groups for each synthesis (item #5)).	10-11
	13b	Describe any methods required to prepare the data for presentation or synthesis, such as handling of missing summary statistics, or data conversions.	10-11
	13c	Describe any methods used to tabulate or visually display results of individual studies and syntheses.	10-11
	13d	Describe any methods used to synthesize results and provide a rationale for the choice(s). If meta-analysis was performed, describe the model(s), method(s) to identify the presence and extent of statistical heterogeneity, and software package(s) used.	10-11
	13e	Describe any methods used to explore possible causes of heterogeneity among study results (e.g. subgroup analysis, meta-regression).	11

Section and Topic	Item #	Checklist item	Location where item is reported
	13f	Describe any sensitivity analyses conducted to assess robustness of the synthesized results.	11
Reporting bias assessment	14	Describe any methods used to assess risk of bias due to missing results in a synthesis (arising from reporting biases).	11
Certainty assessment	15	Describe any methods used to assess certainty (or confidence) in the body of evidence for an outcome.	11
**RESULTS**			
Study selection	16a	Describe the results of the search and selection process, from the number of records identified in the search to the number of studies included in the review, ideally using a flow diagram.	5
	16b	Cite studies that might appear to meet the inclusion criteria, but which were excluded, and explain why they were excluded.	5 and supplement table
Study characteristics	17	Cite each included study and present its characteristics.	5 and supplement table
Risk of bias in studies	18	Present assessments of risk of bias for each included study.	5 and supplementary figure
Results of individual studies	19	For all outcomes, present, for each study: (a) summary statistics for each group (where appropriate) and (b) an effect estimate and its precision (e.g. confidence/credible interval), ideally using structured tables or plots.	5-6 and supplementary figure

Section and Topic	Item #	Checklist item	Location where item is reported
Results of syntheses	20a	For each synthesis, briefly summarise the characteristics and risk of bias among contributing studies.	5-6 and supplementary figure
	20b	Present results of all statistical syntheses conducted. If meta-analysis was done, present for each the summary estimate and its precision (e.g. confidence/credible interval) and measures of statistical heterogeneity. If comparing groups, describe the direction of the effect.	5-6 and supplementary figure
	20c	Present results of all investigations of possible causes of heterogeneity among study results.	5-6
	20d	Present results of all sensitivity analyses conducted to assess the robustness of the synthesized results.	6
Reporting biases	21	Present assessments of risk of bias due to missing results (arising from reporting biases) for each synthesis assessed.	6 and figure 2
Certainty of evidence	22	Present assessments of certainty (or confidence) in the body of evidence for each outcome assessed.	6 and supplementary table
**DISCUSSION**			
Discussion	23a	Provide a general interpretation of the results in the context of other evidence.	7-9
	23b	Discuss any limitations of the evidence included in the review.	7-9
	23c	Discuss any limitations of the review processes used.	7-9
	23d	Discuss implications of the results for practice, policy, and future research.	8-9
**OTHER INFORMATION**			
Registration and	24a	Provide registration information for the review, including register name and registration number, or state that the review was not registered.	9

Section and Topic	Item #	Checklist item	Location where item is reported
protocol	24b	Indicate where the review protocol can be accessed, or state that a protocol was not prepared.	9
	24c	Describe and explain any amendments to information provided at registration or in the protocol.	NA
Support	25	Describe sources of financial or non-financial support for the review, and the role of the funders or sponsors in the review.	11
Competing interests	26	Declare any competing interests of review authors.	11
Availability of data, code and other materials	27	Report which of the following are publicly available and where they can be found: template data collection forms; data extracted from included studies; data used for all analyses; analytic code; any other materials used in the review.	11

From: Page MJ, McKenzie JE, Bossuyt PM, Boutron I, Hoffmann TC, Mulrow CD, et al. The PRISMA 2020 statement: an updated guideline for reporting systematic reviews. BMJ 2021;372:n71. doi: 10.1136/bmj.n71. This work is licensed under CC BY 4.0. To view a copy of this license, visit https://creativecommons.org/licenses/by/4.0/

Supplementary Table 4 Descriptive characteristics of included studies

Study	Design	Country	Participant characteristics			Intervention characteristics			Comparator	Measure
			Sample (male)	Population	Age (SD)	System or device	Frequency & duration	Session length		
Li 2011	NRCT	China	122 (65)	Children with cancer	11.89 (2.22)	PlayMotion	5 sessions/week for 1 week	30 min	Usual care (nursing care)	CES-DC
Rendon 2012	RCT	US	40 (14)	Older adults	84.50 (5.41)	Wii Fit	3 sessions/week for 6 weeks	35-45 min	No intervention	GDS
Wi 2013	RCT	South Korea	40 (0)	Older women with osteoarthritis	75.28 (5.09)	Xbox 360	3 sessions/week for 4 weeks	30 min	Usual care (general physical therapy)	GDS
Chao 2014	NRCT	US	32 (8)	Assisted living residents	85.19 (6.47)	Wii Fit	2 sessions/week for 4 weeks	60 min	Educational session	GDS
Meldrum 2015	RCT	Ireland	71 (27)	Adults with UVL	54.10 (14.97)	Wii Fit Plus	5 sessions/week for 6 weeks	15 min	Vestibular rehabilitation	HADS
Schoene 2015	RCT	Australia	90 (30)	Older adults	81.50 (7.00)	Step pad	3 sessions/week for 16 weeks	20 min	No intervention (brochure)	PHQ
Shin 2015	RCT	South Korea	32 (26)	Stroke patients	53.95 (12.44)	RehabMaster™	5 sessions/week for 4 weeks	30 min	Usual care (conventional occupational therapy)	HAMD
Song 2015	RCT	South Korea	40 (22)	Stroke patients	50.74 (28.87)	Xbox 360	5 sessions/week for 8 weeks	30 min	Bicycle training	BDI
Eggenberger 2016	RCT	Switzerland	33 (12)	Older adults	74.90 (6.90)	Impact Dance Platform	3 sessions/week for 8 weeks	30 min	Balance and stretching training	GDS
Levy 2016	RCT	France	16 (6)	Older adults	70.76 (15.23)	PlayStation	1 session/week for 12 weeks	15 min	No intervention	BDI

Supplementary Table 4 (continued)

Study	Design	Country	Participant characteristics			Intervention characteristics			Comparator	Measure
			Sample (male)	Population	Age (SD)	System or device	Frequency & duration	Session length		
Collado-Mateo 2017	RCT	Spain	83 (0)	Women with fibromyalgia	52.50 (9.20)	Kinect	2 sessions/week for 8 weeks	60 min	No intervention	FIQ
Monteiro-Junior 2017	RCT	Brazil	18 (6)	Institutionalized older adults	85.00 (6.47)	Wii	2 sessions/week for 6-8 weeks	35-45 min	Physical exercise	GDS
Ruivo 2017	RCT	Ireland	32 (26)	Cardiac rehabilitation patients	59.90 (10.20)	Wii	2 sessions/week for 6 weeks	60 min	Physical exercise with music video	HADS
Thomas 2017	RCT	UK	30 (3)	Adults with MS	49.30 (8.70)	Wii	2 sessions/week for 6 months	27 min	Usual care (standard clinical practice)	HADS
Ferraz 2018	RCT	Brazil	62 (37)	Older adults with PD	68.41 (5.55)	Xbox 360	3 sessions/week for 8 weeks	50 min	Functional training (rehabilitation) or bicycle training	GDS
Gomes 2018	RCT	Brazil	30 (2)	Older adults	84.00 (6.00)	Wii Fit Plus	2 sessions/week for 7 weeks	50 min	Usual care (general physical therapy)	GDS
Rodrigues 2018	NRCT	Brazil	47 (0)	Older women	70.24 (4.83)	Xbox 360	3 sessions/week for 12 weeks	40 min	No intervention	GDS
Andrade 2019	NRCT	Brazil	140 (59)	Children	9.41 (0.68)	Xbox	3 sessions/week for 1 week	40 min	Usual physical education class	BRUMS
Maynard 2019	RCT	Brazil	40 (22)	Hemodialysis patients	46.45 (13.64)	Wii Fit Plus	3 sessions/week for 12 weeks	45 min	No intervention	CES-D

Supplementary Table 4 (continued)

Study	Design	Country	Participant characteristics			Intervention characteristics			Comparator	Measure
			Sample (male)	Population	Age (SD)	System or device	Frequency & duration	Session length		
Stanmore 2019	NRCT	UK	106 (23)	Assisted living residents	77.85 (9.44)	Kinect	2 sessions/week for 12 weeks	14.4 min	Usual care and exercise	GDS
Tollár 2019	RCT	Hungary	74 (46)	Older adults with PD	69.39 (4.51)	Xbox 360	5 sessions/week for 5 weeks	60 min	Waiting list control or bicycle training	BDI
Cano-Mañas 2020	RCT	Spain	48 (23)	Stroke patients	63.13 (10.38)	Xbox 360	3 sessions/week for 8 weeks	20 min	Usual care (physical therapy, occupational therapy)	EQ-5D
Carvalho 2020	RCT	Brazil	35 (0)	Women with fibromyalgia	51.33 (13.40)	Wii	3 sessions/week for 7 weeks	60 min	Usual care (chain muscle stretching)	FIQ
Cicek 2020	NRCT	Turkey	44 (22)	Older adults	73.68 (5.50)	Wii Fit Plus	2 sessions/week for 8 weeks	30 min	No intervention or physical exercise	HRSD
García-Bravo 2020	RCT	Spain	20 (NR)	Adults with IHD	51.20 (8.82)	Xbox One	2 sessions/week for 8 weeks	60 min	Resistance exercises	BDI
Lin 2020	RCT	China	80 (39)	Adults with knee OA	57.00 (16.29)	Hot Plus	3 sessions/week for 4 weeks	20 min	Therapeutic exercise	HADS
Ozdogar 2020	RCT	Turkey	60 (16)	Adults with MS	40.1 (40.7)	Xbox One	1 session/week for 8 weeks	45 min	No intervention	BDI
Rica 2020	RCT	Brazil	50 (0)	Older women	Over 60	Xbox 360	3 sessions/week for 12 weeks	60 min	Board games	BDI
Tollár 2020	RCT	Hungary	68 (7)	Adults with MS	47.00 (5.95)	Xbox 360	5 sessions/week for 5 weeks	60 min	Usual care (waiting list) or physical exercise	BDI

Supplementary Table 4 (continued)

Study	Design	Country	Participant characteristics			Intervention characteristics			Comparator	Measure
			Sample (male)	Population	Age (SD)	System or device	Frequency & duration	Session length		
Yunus 2020	RCT	Malaysia	36 (5)	University students	22.89 (1.06)	Xbox 360	3 sessions/week for 6 weeks	30 min	No intervention	DASS
Zhou 2020	RCT	US	73 (33)	Hemodialysis patients	64.50 (8.70)	LEGSys™	3 sessions/week for 4 weeks	30 min	Foot rotation exercise，intradialytic exercise	CES-D
Jahouh 2021	NRCT	Spain	80 (35)	Institutionalized elderly	84.15 (8.70)	Wii	2-3 sessions/week for 8 weeks	40-45 min	Usual care (physical therapy, occupational therapy, and gymnastics sessions)	GDS
Khushnood 2021	RCT	Pakistan	83 (51)	Older adults	Over 60	Wii Fit	2 sessions/week for 8 weeks	45 min	Balance training exercises	EQ-5D
Liu 2021	RCT	US	36 (15)	University students	23.89 (3.57)	PlayStation and VirZoom	2 sessions/week for 4 weeks	60 min	No intervention	BDI
Polat 2021	RCT	Turkey	40 (0)	Women with fibromyalgia	44.80 (8.05)	Xbox	3 sessions/week for 4 weeks	15 min	Bicycle and conventional training	HADS
Swinnen 2021	RCT	Switzerland	45 (10)	Adults with major NCD	84.99 (6.00)	Senso	3 sessions/week for 8 weeks	15 min	Watch music videos	CSDD
Benitez-Lugo 2022	RCT	Spain	46 (14)	Older adults	72.54 (5.82)	Wii	2 sessions/week for 8 weeks	30 min	Usual care (memory workshops and joint mobility workshops)	GDS
Moret 2022	RCT	Italy	57 (15)	Older adults	70.47 (3.72)	Xbox 360	3-4 sessions/week for 2-3 weeks	45 min	No intervention	BDI

Supplementary Table 4 (continued)

Study	Design	Country	Participant characteristics			Intervention characteristics			Comparator	Measure
			Sample (male)	Population	Age (SD)	System or device	Frequency & duration	Session length		
Ozdogar 2022	RCT	Turkey	30 (9)	Adults with MS	37.60 (11.60)	Xbox One	Once daily for 7.87 (1.90) days	45 min	Rehabilitation program (balance, gait, upper extremity, and core stability exercises)	HADS
Zheng 2022	RCT	China	38 (9)	Older adults with dementia	83.07 (5.70)	Xbox 360	5 sessions/week for 8 weeks	60 min	Usual care	CSDD
Cavusoglu 2023	RCT	Turkey	16 (11)	Pediatric patients with CKD	11.31 (3.49)	Wii	2 sessions/week for 6 weeks	40 min	Fun physical exercise (with cartoons)	CDI
Lee 2023	RCT	South Korea	57 (31)	Community-dwelling older adults	79.73 (3.35)	Switch	3 sessions/week for 8 weeks	50 min	No intervention	GDS
Manser 2023	RCT	Switzerland	16 (9)	Older adults with mild NCD	77.57 (9.99)	Senso (Flex)	5 sessions/week for 12 weeks	21 min	Usual care (medication, physiotherapy)	DASS
Ozdogar 2023	RCT	Turkey	65 (41)	Adults with MS	40.62 (14.93)	Xbox One	2 sessions/week for 8 weeks	45 min	Usual care (No physical activity)	HADS
Turon-Skrzypinska 2023	RCT	Poland	85 (58)	Hemodialysis patients	60.3 (16.58)	NefroVR	3 sessions/week for 3 months	20 min	No intervention	BDI
Ünver 2023	RCT	Turkey	14 (7)	Older adults	> 60	Kinect	3 sessions/week for 3 weeks	30 min	Usual care	GDS
Abbas 2024	RCT	Lebanon	32 (29)	Traumatic LLAs	27.63 (5.97)	Xbox	3 sessions/week for 6 weeks	25 min	Rehabilitation exercise	BDI
Alves 2024	RCT	Portugal	70 (45)	Cancer patients	61.46 (10.65)	Wii Fit	Once daily for 6 days	15-30 min	Usual care	HADS

Supplementary Table 4 (continued)

Study	Design	Country	Participant characteristics			Intervention characteristics			Comparator	Measure
			Sample (male)	Population	Age (SD)	System or device	Frequency & duration	Session length		
Ferreira 2024	NRCT	Portugal	78 (NR)	Community-dwelling older adults	72.50 (6.27)	PEPE	3 sessions/week for 12 weeks	60 min	No intervention or physical and cognitive exercise	GDS
Massah 2024	RCT	Iran	40 (22)	Adults with CLBP	31.00 (1.22)	Xbox 360	A single session	30-45 min	Physical exercise	BRUMS
Nuic 2024	RCT	France	50 (31)	Adults with PD	66.70 (7.74)	Kinect	2-3 sessions/week for 6-9 weeks	15-45 min	Computer games	HADS
Swinnen 2024	RCT	Switzerland	18 (1)	Adults with major NCD	83.31 (6.75)	VITAAL	3 sessions/week for 12 weeks	30 min	Physical exercise	CSDD
Sturnieks 2024	RCT	Australia	769 (220)	Community-dwelling older people	72.57 (5.56)	Smart±step	NR	NR	No intervention or cognitive training	PHQ
Wong 2024	RCT	China	202 (NR)	Community-dwelling older adults	78.80 (7.80)	NR	12 sessions in total for 18 weeks	NR	Usual care (health talks and physical activity class; play the same games)	GDS
Yuenyongchaiwat 2024	RCT	Thailand	60 (35)	Adults with CAD or VD	63.82 (9.10)	Toucher	Once daily for 8.64 (2.45) days	NR	Physical therapy	PHQ
Hsieh 2025	RCT	China	70 (26)	Adults with CLBP	59.65 (11.55)	Kinect	6 sessions in total for 2 weeks	15 min	Physical exercise	HADS
Klompstra 2025	RCT	Sweden	104 (66)	Adults with heart failure	71.00 (12.00)	Wii	5 sessions/week for 3 months	Over 30 min	No intervention or medical yoga	HADS
Sakamoto 2025	RCT	Japan	10 (4)	Adults with glioma	57.81(27.64)	Wii	3 sessions/week for 80.84 (21.08) days	20 min	Physiotherapy	HADS

Supplementary Table 5 Abbreviations

BDI	Beck Depression Inventory
BRUMS	Brunel Mood Scale
CAD	Coronary Artery Disease
CDI	Child Depression Inventory
CES-D	Center for Epidemiologic Studies Depression Scale
CES-DC	Center for Epidemiologic Studies Depression Scale for Children
CKD	Chronic Kidney Disease
CLBP	Chronic Low Back Pain
CR	Cardiac Rehabilitation
CSDD	Cornell Scale for Depression in Dementia
DASS	Depression, Anxiety, and Stress Scale
EQ-5D	EuroQoL 5D
FIQ	Fibromyalgia Impact Questionnaire
GDS	Geriatric Depression Scale
HADS	Hospital Anxiety and Depression Scale
HAMD	Hamilton Depression Rating Scale
HRSD	Hamilton Rating Scale for Depression
IHD	Ischemic Heart Disease
LLAs	Lower Limb Amputees
MS	Multiple Sclerosis
NCD	Neurocognitive Disorder
NRCT	Non-randomized controlled trials
NR	Not reported
OA	Osteoarthritis
PD	Parkinson Disease

PEPE	Portable Exergame Platform for Elderly
PHQ	Patient Health Questionnaire
POMS	Profile of Mood States Brief-Form
RCT	Randomized controlled trial
SD	Standardized deviation
SDS	Self-rating Depression Scale
UVL	Unilateral Peripheral Vestibular Loss
VD	Valvular Disease

Supplementary Table 6 Grading of Recommendations Assessment, Development and Evaluation

Quality assessment							No. of patients		Effect	Quality
No. of studies	Design	Risk of bias	Inconsistency	Indirectness	Imprecision	Other considerations	Intervention	Control	SMD (95% CI)	
**Depression (measured with:depression scales; Better indicated by lower values)**										
58	randomized trials	serious[1]	very serious[2]	no serious indirectness	no serious imprecision	none	1787	1827	−0.40 (−0.56 to −0.25)	⊕OOO VERY LOW

[1] Downgraded one level due to serious risk of bias, as the majority of included studies were assessed as having moderate risk of bias.

[2] Downgraded two levels due to substantial statistical heterogeneity ($I^2 > 75\%$). Through subgroup analyses, we found that this heterogeneity could be partially attributed to variations in control group designs and differences in intervention frequency. These methodological differences across studies may explain the observed statistical heterogeneity

Supplementary Figure 1 PRISMA selection flow diagram

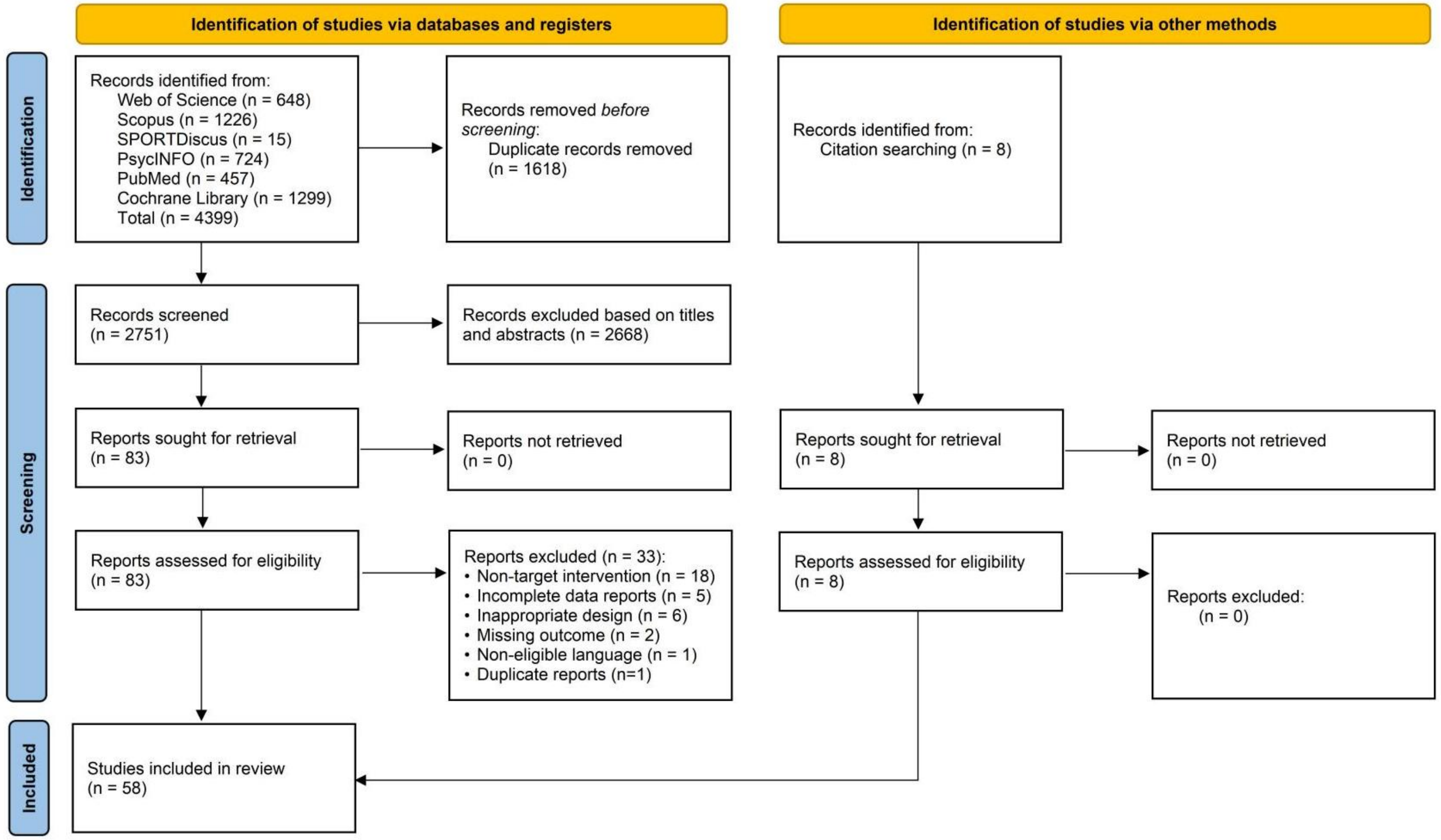

Source: Page MJ, et al. BMJ 2021;372:n71. doi: 10.1136/bmj.n71.

Supplementary Figure 2 Risk of bias assessment for each included randomized study

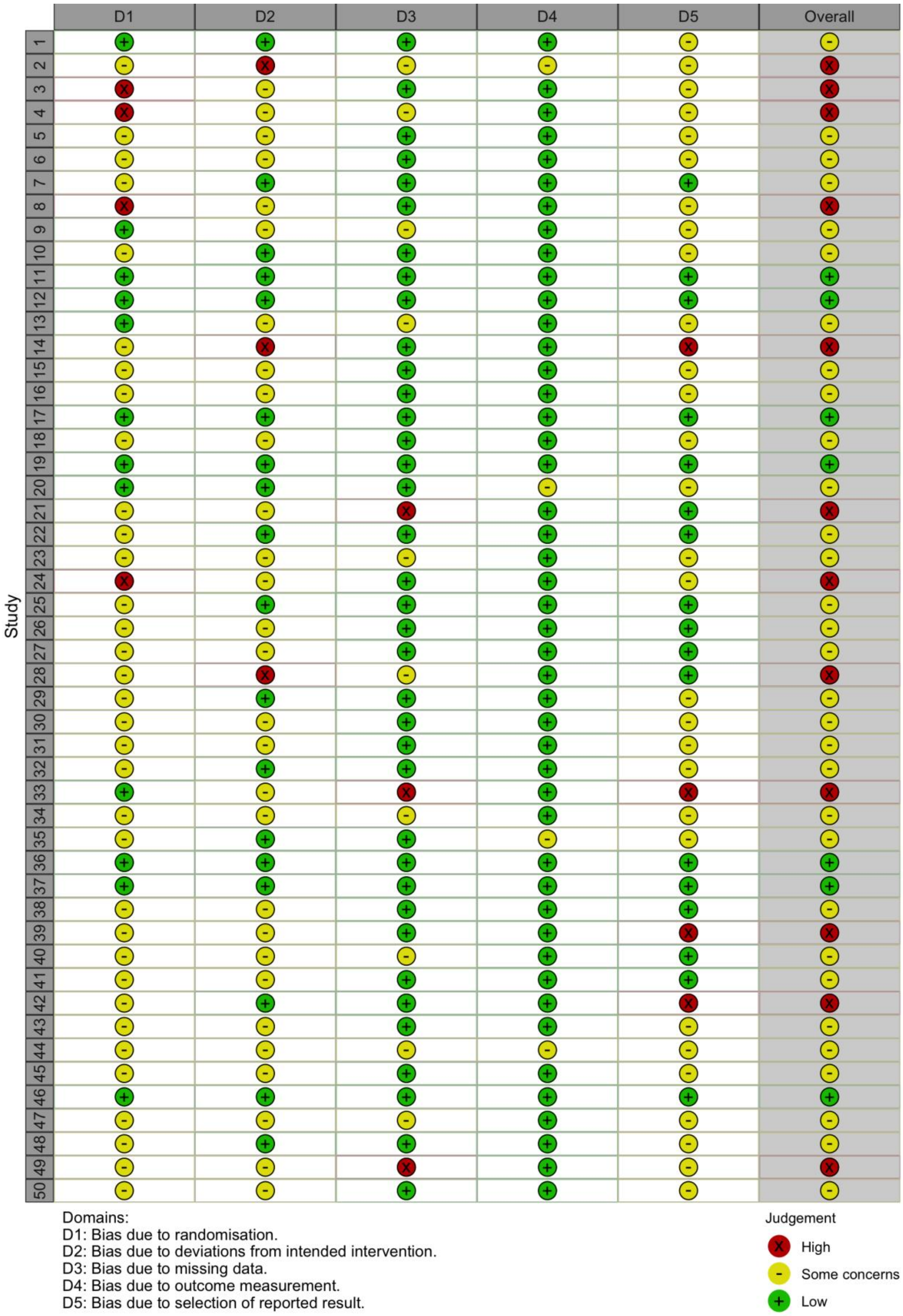

This figure was generated using R version 4.4.2.

No.	Title
**1**	Effect of adding virtual reality training to traditional exercise program on pain, mental status and psychological status in unilateral traumatic lower limb amputees: a randomized controlled trial
**2**	Exergame-based rehabilitation for cancer patients undergoing abdominal surgery: effects on pain, anxiety, depression, and fatigue - a pilot study
**3**	Effectiveness of feedback-based technology on physical and cognitive abilities in the elderly
**4**	Effects of video-game based therapy on balance, postural control, functionality, and quality of life of patients with subacute stroke: a randomized controlled trial
**5**	Effects of exergames in women with fibromyalgia: a randomized controlled study
**6**	Comparison of effectiveness of Nintendo Wii-based exergaming and home-based fun video exercises in pediatric patients with chronic kidney disease
**7**	Effects of exergames on quality of life, pain, and disease effect in women with fibromyalgia: a randomized controlled trial
**8**	Exergame and balance training modulate prefrontal brain activity during walking and enhance executive function in older adults
**9**	The effects of functional training, bicycle exercise, and exergaming on walking capacity of elderly patients with Parkinson disease: a pilot randomized controlled single-blinded trial
**10**	Effects of virtual reality on cardiac rehabilitation programs for ischemic heart disease: a randomized pilot clinical trial
**11**	Feasibility, safety, acceptability, and functional outcomes of playing nintendo wii fit plustm for frail older adults: a randomized feasibility clinical trial
**12**	Short-term effects of exergaming on patients with chronic low back pain: a single-blind randomized controlled trial
**13**	Role Wii Fit exer-games in improving balance confidence and quality of life in elderly population
**14**	Effects of exergaming and yoga on exercise capacity and physical and mental health in heart failure patients: a randomized sub-study
**15**	Home-based exergame program to improve physical function, fall efficacy, depression and quality of life in community-dwelling older adults: a randomized controlled trial
**16**	Fear of falling: efficacy of virtual reality associated with serious games in elderly people
**17**	Active video games for knee osteoarthritis improve mobility but not WOMAC score: a randomized controlled trial
**18**	Effects of virtual reality exercise on promoting physical activity and health among college students: a 4-week randomized controlled trial
**19**	Feasibility, usability, and acceptance of “Brain-It”—a newly developed exergame-based training concept for the secondary prevention of mild neurocognitive disorder: a pilot randomized controlled trial
**20**	Comparison of the acute effects of virtual reality exergames and core stability exercises on cognitive factors, pain, and fear avoidance beliefs in people with chronic nonspecific low back pain
**21**	Effects of exercise training combined with virtual reality in functionality and health-related quality of life of patients on hemodialysis
**22**	Effectiveness of conventional versus virtual reality-based balance exercises in vestibular rehabilitation for unilateral peripheral vestibular loss: results of a randomized controlled trial
**23**	Virtual reality–based physical exercise with exergames (Physex) improves mental and physical health of institutionalized older adults
**24**	Effects of exergames on mood and cognition in healthy older adults: a randomized pilot study
**25**	Home-based exergaming to treat gait and balance disorders in patients with Parkinson's disease: a phase ii randomized controlled trial
**26**	Effect of video-based exergaming on arm and cognitive function in persons with multiple sclerosis: a randomized controlled trial
**27**	Effects and safety of exergaming in persons with multiple sclerosis during corticosteroid treatment: a pilot study
**28**	Effect of exergaming in people with restless legs syndrome with multiple sclerosis: a single-blind randomized controlled trial

**29**	The effect of virtual reality exercises on pain, functionality, cardiopulmonary capacity and quality of life in fibromyalgia syndrome: a randomized, single-blind, controlled study
**30**	The effect of virtual reality gaming on dynamic balance in older adults
**31**	Effects of a Kinect-based physical training program on body composition, functional fitness and depression in institutionalized older adults
**32**	In-class active video game supplementation and adherence to cardiac rehabilitation
**33**	Interactive cognitive-motor step training improves cognitive risk factors of falling in older adults–a randomized controlled trial
**34**	Effects of game-based virtual reality on health-related quality of life in chronic stroke patients: a randomized, controlled study
**35**	Effect of virtual reality games on stroke patients' balance, gait, depression, and interpersonal relationships
**36**	The effectiveness and cost-effectiveness of strength and balance exergames to reduce falls risk for people aged 55 years and older in UK assisted living facilities: a multi-centre, cluster randomised controlled trial
**37**	Exergame and cognitive training for preventing falls in community-dwelling older people: a randomized controlled trial
**38**	The efficacy of exergaming in people with major neurocognitive disorder residing in long-term care facilities: a pilot randomized controlled trial
**39**	The feasibility of a stepping exergame prototype for older adults with major neurocognitive disorder residing in a long-term care facility: a mixed methods pilot study
**40**	Mii-Vitalise: a pilot randomised controlled trial of a home gaming system (Nintendo Wii) to increase activity levels, vitality and well-being in people with multiple sclerosis
**41**	Vastly different exercise programs similarly improve Parkinsonian symptoms: a randomized clinical trial
**42**	Exercise effects on multiple sclerosis quality of life and clinical-motor symptoms
**43**	Impact of virtual reality exercises on anxiety and depression in hemodialysis
**44**	The effect of virtual reality application on pain, functional independence, and depression in the older adults: a pilot study
**45**	Clinical feasibility of exercise game for depression treatment in older women with osteoarthritis: a pilot study
**46**	The effect of young people-assisted, individualized, motion-based video games on physical, cognitive, and social frailty among community-dwelling older adults with frailty: randomized controlled trial
**47**	Effects of virtual exercise on cardio-pulmonary performance and depression in cardiac rehabilitation phase i: a randomized control trial
**48**	Investigating the feasibility of exergame on sleep and emotion among university students
**49**	The effect of young people-assisted, individualized, motion-based video games on physical, cognitive, and social frailty among community-dwelling older adults with frailty: randomized controlled trial
**50**	Application of wearables to facilitate virtually supervised intradialytic exercise for reducing depression symptoms

Supplementary Figure 3 Summary of risk of bias assessment for all included randomized studies

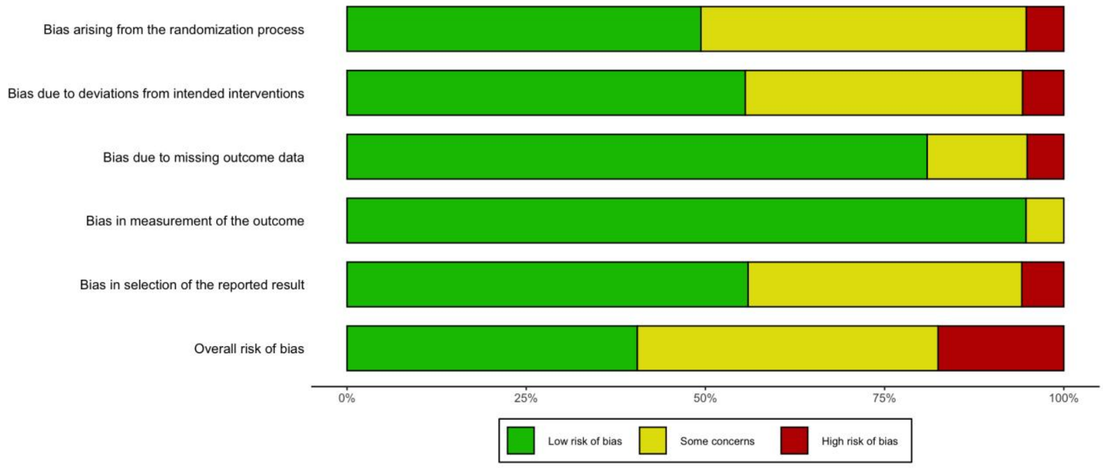

This figure was generated using R version 4.4.2.

Supplementary Figure 4 Risk of bias assessment for each included non-randomized study

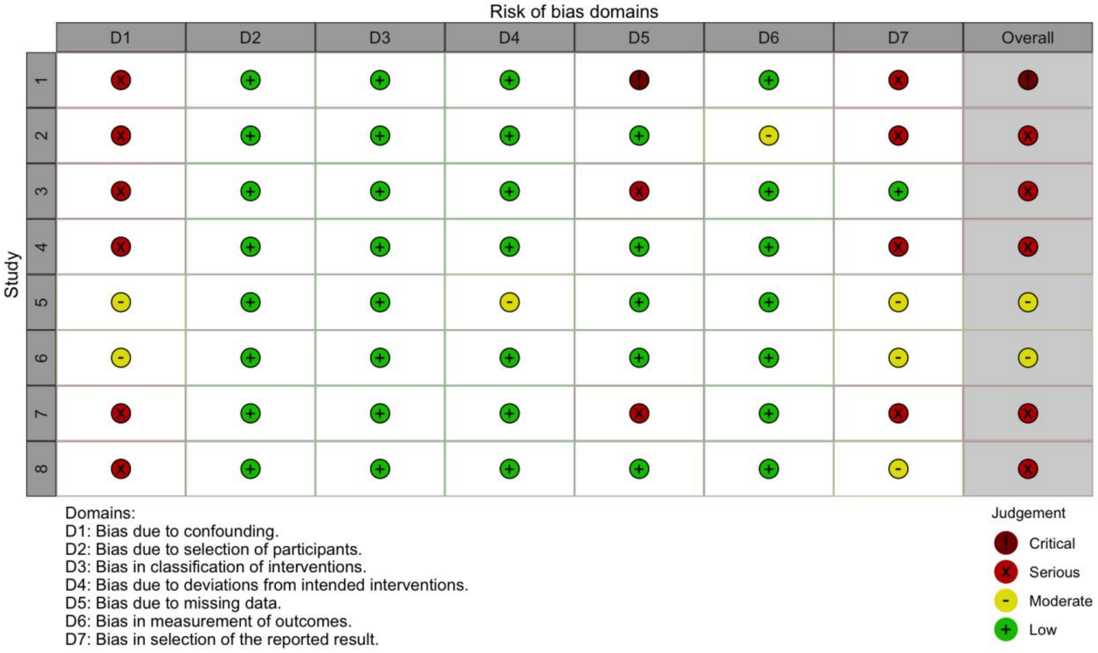

This figure was generated using R version 4.4.2.

No.	Title
1	Acute effect of exergames on children's mood states during physical education classes
2	Physical and psychosocial effects of Wii Fit exergames use in assisted living residents: a pilot study
3	Interactive video game-based approaches improve mobility and mood in older adults: a nonrandomized, controlled trial
4	Effects of multimodal exercise with augmented reality on cognition in community-dwelling older adults
5	Impact of an intervention with Wii video games on the autonomy of activities of daily living and psychological–cognitive components in the institutionalized elderly
6	The effectiveness of therapeutic play, using virtual reality computer games, in promoting the psychological well-being of children hospitalised with cancer
7	Effects of dance exergaming on depressive symptoms, fear of falling, and musculoskeletal function in fallers and nonfallers community-dwelling older women
8	Could video game-based physical rehabilitation substitute for conventional physiotherapy in patients with glioma? A proof-of-concept study

Supplementary Figure 5 Summary of risk of bias assessment for all included non-randomized studies

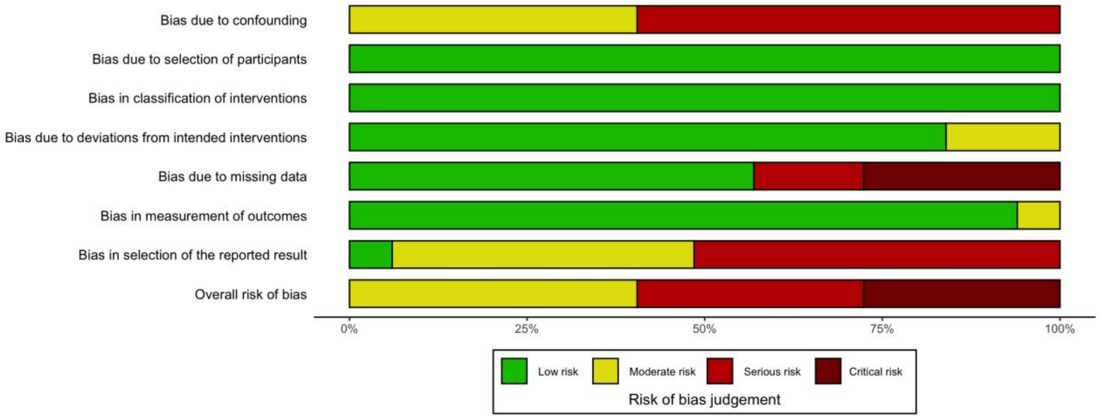

This figure was generated using R version 4.4.2.

Supplementary Figure 6 Forest plot for the effect of exergaming for depression

Study	Intervention group Total	Intervention group Mean change	Intervention group SD	Control group Total	Control group Mean change	Control group SD	Hedges' g[95% CI]
Yuenyongchaiwat 2024	25	1.68	0.92	22	4	0.98	−2.41 [−3.16, −1.65]
Rica 2020	17	−5.94	2.76	33	0.84	3.46	−2.06 [−2.77, −1.35]
Lee 2023	28	−2.68	1.93	29	0.21	0.94	−1.89 [−2.51, −1.26]
Unver 2023	7	−2.57	1.66	7	−0.28	0.6	−1.72 [−2.94, −0.49]
Benitez−Lugo 2022	26	−2.61	1.96	20	0	0.8	−1.64 [−2.31, −0.96]
Song 2015	20	−7.1	3.33	20	−2.1	2.98	−1.55 [−2.26, −0.84]
Swinnen 2021	23	−4	5.67	22	4	5.91	−1.36 [−2.01, −0.71]
Tollar 2019−1	25	−2.5	2.28	25	0.1	1.98	−1.20 [−1.80, −0.60]
Zheng 2022	18	−8.06	5.34	20	1.85	10.01	−1.19 [−1.88, −0.50]
Monteiro−Junior 2017	9	−2	3	9	1	3	−0.95 [−1.93, 0.02]
Li 2011	52	−1.71	6.03	70	3.87	6	−0.92 [−1.30, −0.55]
Rodrigues 2018	22	−2.5	2.24	25	−0.4	2.5	−0.87 [−1.47, −0.27]
Carvalho 2020	14	−4.64	2.5	14	−2.64	2.46	−0.78 [−1.55, −0.01]
Wi 2013	20	−1.7	2	20	−0.2	2.1	−0.72 [−1.36, −0.08]
Levy 2016	9	−3	10.79	7	3.25	2.75	−0.71 [−1.72, 0.31]
Jahouh 2021	40	−1.3	3.22	40	0.73	2.99	−0.65 [−1.10, −0.20]
Cicek 2020−1	16	−1.87	3.72	14	0.29	3.54	−0.58 [−1.31, 0.15]
Cano−Manas 2020	25	−0.69	0.6	23	−0.28	0.81	−0.57 [−1.15, 0.01]
Liu 2021	17	−2.7	5.11	19	0.53	6.51	−0.54 [−1.20, 0.13]
Moret 2022	38	−2.69	5.4	19	0.16	5.3	−0.52 [−1.08, 0.04]
Ozdogar 2020−1	20	−3.9	7.1	20	−0.85	4.59	−0.50 [−1.13, 0.13]
Ozdogar 2023	23	−3.81	9.54	29	−0.38	3.97	−0.48 [−1.04, 0.07]
Turon−Skrzypinska 2023	39	−1.22	6.36	46	2.55	9.41	−0.46 [−0.89, −0.03]
Manser 2023	8	−2	3.41	6	0	4.95	−0.45 [−1.53, 0.62]
Khushnood 2021	42	−0.74	0.56	41	−0.51	0.51	−0.43 [−0.86, 0.01]
Yunus 2020	18	−1.23	3.06	18	−0.05	2.87	−0.39 [−1.05, 0.27]
Cicek 2020−2	16	−1.87	3.72	14	−0.57	2.87	−0.38 [−1.10, 0.35]
Maynard 2019	20	−5.6	7.56	20	−2.7	8.71	−0.35 [−0.97, 0.28]
Polat 2021	20	−1.85	2.59	20	−0.9	2.91	−0.34 [−0.96, 0.29]
Ozdogar 2020−2	20	−3.9	7.1	17	−1.9	5.1	−0.31 [−0.96, 0.34]
Alves 2024	35	−0.5	3.42	35	0.6	3.55	−0.31 [−0.78, 0.16]
Ferreira 2024−1	21	−1.38	2.95	22	−0.46	3.06	−0.30 [−0.90, 0.30]
Ferreira 2024−2	21	−1.38	2.95	21	−0.67	1.65	−0.29 [−0.90, 0.32]
Schoene 2015	39	0.5	4.59	42	1.71	3.69	−0.29 [−0.73, 0.15]
Collado−Mateo 2017	42	−0.5	3.12	41	0.37	3.05	−0.28 [−0.71, 0.15]
Thomas 2017	15	−0.56	2.97	14	0.26	2.8	−0.28 [−1.01, 0.46]
Klompstra 2025−1	27	−0.06	2.6	31	0.59	2.23	−0.27 [−0.78, 0.25]
Chao 2014	15	−0.67	2.27	15	−0.06	2.3	−0.26 [−0.98, 0.46]
Tollar 2019−2	25	−2.5	2.28	24	−2.1	2.35	−0.17 [−0.73, 0.39]
Meldrum 2015	36	−1.83	3.55	35	−1.21	3.96	−0.16 [−0.63, 0.30]
Swinnen 2024	7	−0.8	2.72	11	−0.1	4.8	−0.16 [−1.11, 0.79]
Hsieh 2025	35	−1.47	3.08	35	−1.01	2.9	−0.15 [−0.62, 0.32]
Massah 2024	20	−1	1.82	20	−0.7	2.44	−0.14 [−0.76, 0.48]
Wong 2024	101	−1.5	3.62	101	−1.16	3.98	−0.09 [−0.36, 0.19]
Klompstra 2025−2	27	−0.06	2.6	30	0.16	2.74	−0.08 [−0.60, 0.44]
Ozdogar 2022	12	−0.87	3.56	14	−0.6	3.42	−0.08 [−0.85, 0.70]
Sturnieks 2024	252	0.1	2.72	255	0.3	3.75	−0.06 [−0.24, 0.11]
Shin 2015	16	−2.42	4.75	16	−2.47	5.76	0.01 [−0.68, 0.70]
Eggenberger 2016	19	0.06	3.35	14	−0.07	2.6	0.04 [−0.65, 0.73]
Gomes 2018	15	0.2	2.91	15	0.07	3.16	0.04 [−0.67, 0.76]
Tollar 2020−1	14	−0.2	2.67	12	−0.4	2.97	0.07 [−0.70, 0.84]
Lin 2020	40	0.2	3.8	40	−0.3	4.31	0.12 [−0.32, 0.56]
Ruivo 2017	16	−0.55	4.06	16	−1.09	3.54	0.14 [−0.56, 0.83]
Garcia−Bravo 2020	10	−1	24.69	10	−4	6.51	0.16 [−0.72, 1.04]
Stanmore 2019	43	−0.2	1.3	49	−0.42	1.21	0.17 [−0.24, 0.58]
Andrade 2019	66	−0.07	1.47	72	−0.3	0.9	0.19 [−0.15, 0.52]
Zhou 2020	37	−4.8	7.07	36	−6.9	10.76	0.23 [−0.23, 0.69]
Sakamoto 2025	4	2.67	2.67	6	1.34	6.06	0.24 [−1.03, 1.51]
Abbas 2024	16	−2.25	5.46	16	−4.09	7.47	0.27 [−0.42, 0.97]
Tollar 2020−2	14	−0.2	2.67	14	−1	2.75	0.29 [−0.46, 1.03]
Ferraz 2018−2	20	1	1.6	22	0.5	1.19	0.35 [−0.26, 0.96]
Cavusoglu 2023	8	−2.25	3.15	8	−3.75	3.69	0.41 [−0.58, 1.40]
Rendon 2012	20	1.09	4.37	20	−1.04	4.01	0.50 [−0.13, 1.13]
Nuic 2024	23	0	3.1	25	−1.5	2.5	0.53 [−0.05, 1.10]
Random−Effects Model							−0.40 [−0.56, −0.25]

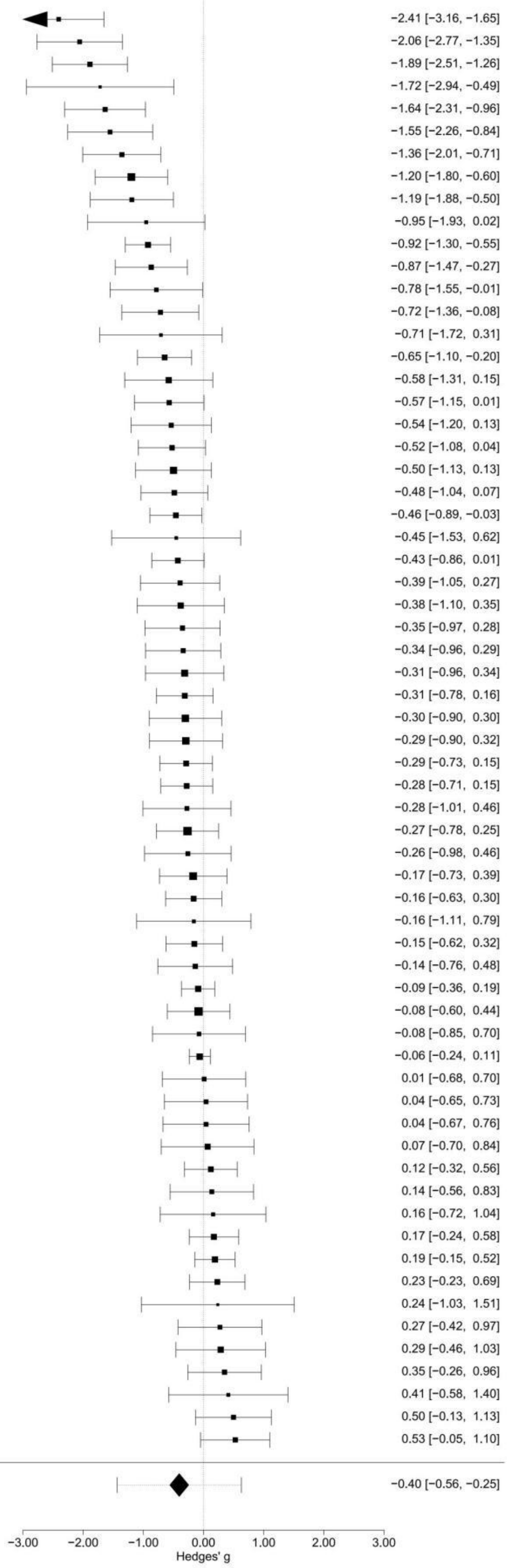

This figure was generated using R version 4.4.2.
